# In situ fNIRS measurements during cognitive behavioral emotion regulation training in rumination-focused therapy: A randomized-controlled trial

**DOI:** 10.1016/j.nicl.2023.103525

**Published:** 2023-10-13

**Authors:** Hendrik Laicher, Isabell Int-Veen, Leonie Woloszyn, Ariane Wiegand, Agnes Kroczek, Daniel Sippel, Elisabeth J. Leehr, Glenn Lawyer, Francesco Albasini, Christian Frischholz, Rainald Mössner, Vanessa Nieratschker, Julian Rubel, Andreas Fallgatter, Ann-Christine Ehlis, David Rosenbaum

**Affiliations:** a Department of Psychiatry and Psychotherapy, University Hospital of Tuebingen, Tübingen Center for Mental Health (TüCMH), Tuebingen, Germany; b German Center for Mental Health (DZPG), partner site Tuebingen, Germany; c Max-Planck Institute of Psychiatry, Munich, Germany; d Institute for Translational, University of Muenster, Muenster, Germany; e Machine Learning Solutions, Luxembourg, Luxembourg; f Psychotherapy Research Unit, Department of Psychology, Osnabrueck University, Osnabrueck, Germany; g LEAD Graduate School & Research Network, University of Tuebingen, Tuebingen, Germany

**Keywords:** functional near-infrared spectroscopy (fNIRS), Emotion Regulation, Major Depression, Psychotherapy, In situ measurements, Repetitive Negative Thinking (RNT)

## Abstract

Repetitive negative thinking (RNT), including rumination, plays a key role in various psychopathologies. Although several psychotherapeutic treatments have been developed to reduce RNT, the neural correlates of those specific treatments and of psychotherapy in general are largely unknown. Functional near-infrared spectroscopy (fNIRS) offers the potential to investigate the neural correlates of psychotherapeutic techniques in situ. Therefore, in this study we investigated the efficacy and neural correlates of a fNIRS adapted Mindfulness-based Emotion Regulation Training (MBERT) for the treatment of depressive rumination in 42 subjects with major depressive disorder (MDD) in a cross-over designed randomized controlled trial. Using psychometric measures, subjective ratings and fNIRS, we analyzed in situ changes in depressive symptom severity, ruminative thoughts and cortical activity in the Cognitive Control Network (CCN). Our results show that MBERT is effective in treating depressive symptoms and rumination. On a neural level, we found consistently higher cortical activation during emotion regulation training compared to control trials in the bilateral inferior frontal gyrus (IFG) and dorsolateral prefrontal cortex (DLPFC). Furthermore, cortical oxygenation decreased from session to session in the bilateral DLPFC. The relevance of the results for the psychotherapeutic treatment of MDD as well as further necessary investigations are discussed.

## Highlights

• Mindfulness-based Emotion Regulation Training is effective to treat depression.
• Mindfulness-based Emotion Regulation Training reduces rumination.
• Self-compassion and self-efficacy are increased through training.
• Active Emotion Regulation induces activation in the cognitive control network.
• Neural activation in this network decreases over the course of training.

## 1 Introduction

With a lifetime prevalence of up to 20 % (Bijl et al., 1998, Ebmeier et al., 2006, Hasin et al., 2018) and probably even rising prevalence rates (Vos et al., 2020a), major depressive disorder (MDD) is considered a leading contributor to the global burden of disease (Vos et al., 2020a, Vos et al., 2020b). As depression is a mood disorder, it seems likely that the core of the psychopathology is related to emotion regulation (ER) difficulties (Durbin & Shafir, 2008). Patients suffering from MDD seem to use adaptive ER strategies less frequently (e.g. reappraisal, problem solving and acceptance) and more frequently maladaptive ones (e.g. suppression, avoidance and rumination) (Barnow et al., 2013, Joormann and Stanton, 2016, Vanderlind et al., 2020). Further, depressed individuals experience prolonged episodes of negative emotions such as sadness and depressed mood in response to negative events (Nolen-Hoeksema et al., 2008, Rosenbaum et al., 2021, Rosenbaum et al., 2018d, Teasdale, 1988). In their reviews, Joormann and Quinn (2014) and Joormann and Stanton (2016) summarize that the difficulties in ER in MDD might interfere with cognitive processes and lead to cognitive biases and deficits in cognitive control.

Up to date, there are several treatment options for MDD with relatively high response rates (Beck & Alford, 2014). The state-of-the-art treatment (American Psychological Association, 2019) combines psychotherapy (Beck and Alford, 2014, Papageorgiou and Wells, 2004) and antidepressant medication (Beck and Alford, 2014, Blackburn et al., 1986, DeRubeis et al., 1999, Shea et al., 1992). However, despite these treatment options, between one to two thirds of patients with MDD experience two or more depressive episodes (Steinert et al., 2014, Vittengl et al., 2007). One risk factor for relapse in MDD is depressive rumination (Michalak et al., 2011, Smith and Alloy, 2009). Originally, rumination was defined by Nolen-Hoeksema as “behavior and thoughts that focus one’s attention on one’s depressive symptoms and on the implications of these symptoms” (p. 569, Nolen-Hoeksema & Morrow, 1991). Moreover, in a broader definition rumination describes a perseverative, highly self-referential, pessimistic and abstract thinking style with little or no goal- and change-orientation (Teismann et al., 2012) and it is conceptualized as a form of repetitive negative thinking, such as worry (McEvoy et al., 2013). While the Response Styles Theory (Nolen-Hoeksema, 1991) assumes that rumination is a common response to negative affect, recent theories assume the relevance of social emotions such as shame and guilt and the experience of stress (O'Connor et al., 2007) as more specific triggers of rumination. Supporting this hypothesis, rumination has been found to be associated with reduced emotional wellbeing, emotion dysregulation (Gross and John, 2003, Slavish and Graham-Engeland, 2015) and higher physiological stress parameters (Ottaviani et al., 2016). Further, rumination interferes with problem solving as well as instrumental behavior and is linked to higher symptom severity, prolonged negative mood states, longer duration of depressive episodes and higher risk of suicide (Eshun, 2000, Ito et al., 2006, Koval et al., 2012, Law and Tucker, 2018, Papageorgiou and Wells, 2004, Smith and Alloy, 2009, Spasojevic and Alloy, 2001).

On a neural level, enhanced activations in areas of the Cognitive Control Network (CCN) and the Default Mode Network (DMN) were found in depressed patients during guided rumination-induction paradigms (Burkhouse et al., 2017, Cooney et al., 2010, Hamilton et al., 2011, Jones et al., 2017, Longe et al., 2010). Further, aberrant functional connectivity between areas of the DMN and the CCN were found (Hamilton et al., 2015, Jacobs et al., 2014, Peters et al., 2016, Rosenbaum et al., 2017, Rosenbaum et al., 2018d, Goldbeck et al., 2019, Lydon-Staley et al., 2019). The CCN, involving areas such as the inferior frontal gyrus (IFG), the dorsolateral prefrontal cortex (DLPFC) and the posterior parietal cortex, plays a role in cognitive control and top-down regulation processes (Cole and Schneider, 2007, Rosenbaum et al., 2016). Concerning emotion regulation, the down-regulation of negative affect in high ruminators has been linked to increased activity in subcortical areas and decreased prefrontal activity in the CCN (Ray et al., 2005, Rosenbaum et al., 2020a, Vanderhasselt et al., 2013). In previous investigations we further found high ruminators to have reduced neural activity in the CCN when confronted with social stress (Henze et al., 2023, Rosenbaum et al., 2018a, Rosenbaum et al., 2018b, Rosenbaum et al., 2021, Rosenbaum et al., 2018d).

Understanding rumination as a result of maladaptive ER (Joormann & Gotlib, 2010), an approach to treat rumination directly might work by increasing adaptive ER, incorporating cognitive-behavioral and mindfulness-based techniques. Indeed, treatments that directly foster ER processes, such as Emotion Regulation Therapy, reduce self-referential processes such as rumination and worry in MDD and generalized anxiety disorder (Mennin et al., 2015, Mennin et al., 2018, Renna et al., 2018). The Emotion Regulation Therapy used in these studies was composed of 16 to 20 sessions and subjects were taught ER strategies via attention regulation and meta-cognitive strategies to enhance their proactive deployment of regulation skills. Mennin et al. (2018) report significantly reduced repetitive negative thoughts in subjects after completion of the therapy. On a neural level, studies using fMRI showed an association of ER with enhanced activation in parts of the CCN such as the DLPFC (Buhle et al., 2014, Burklund et al., 2014, Guendelman et al., 2017). However, the investigated ER strategies in all of these studies were cognitive behavioral. In their meta-analysis, Perestelo-Perez et al. (2017) found mindfulness-based interventions, such as mindfulness-based stress reduction (MBSR, developed by Kabat-Zinn, 2003), to also reduce rumination in patients suffering from MDD. In line with this, recent therapeutic approaches (“third wave CBT”) such as mindfulness-based cognitive therapy (MBCT, Segal et al., 2002) or Acceptance and Commitment Therapy (ACT, Hayes et al., 1999), integrated mindfulness-based elements into classic cognitive behavioral approaches. The effectiveness of such therapeutic approaches in the treatment of MDD – and of rumination in particular – has been demonstrated in several randomized-controlled trial (RCT) studies and systematic reviews (e.g. Chiesa and Serretti, 2011, Clarke et al., 2015, Piet and Hougaard, 2011, Jain et al., 2015). Again, the DLPFC was found to play an important role in these treatments (Chiesa and Serretti, 2011, Vago and Silbersweig, 2012).

Following these first promising results, we developed an eight-session psychotherapeutic training, combining mindfulness-based as well as cognitive behavioral ER therapy: Mindfulness-based Emotion Regulation Training (MBERT). This approach was fNIRS-adapted, allowing in situ measurements of neural processes. To the best of our knowledge, this study is the first to investigate the efficacy and the in situ neural correlates of such a mindfulness-based ER approach in the treatment of MDD. In the study at hand, we compared MBERT in a RCT with treatment as usual (TAU; no additional interventions with unrestricted continuation of existing treatments) in 42 patients suffering from MDD using a cross-over design. Our treatment consisted of a psychoeducative explanation of rumination, its link to negative emotions and impairments in ER, followed by eight psychotherapeutic training sessions practicing cognitive behavioral and mindfulness-based ER strategies under instruction and support by a clinical psychologist/psychotherapist. Each of the eight training sessions consisted of 20 training trials, in which the ER strategies were practiced, and rest trials without any specific (cognitive) task as control condition. To evaluate the efficacy of this treatment approach, we used psychometric (primary outcomes) and subjective (secondary outcomes) rating endpoints. Further, in situ fNIRS measurements were conducted to measure hemodynamic responses in the course of each session (secondary outcome). In general, we hypothesized a reduction in depressive symptoms as well as increases in self-compassion and self-efficacy due to the MBERT compared to TAU (primary outcomes). These hypotheses are derived from the knowledge about the general factors and mechanisms of psychotherapy and of “third wave CBT” in particular, namely enhanced self-compassion (Kuyken et al., 2010, Wilson et al., 2019) and self-efficacy (Bandura and Adams, 1977, Bandura, 1978). During each session as well as across sessions, reductions in subjective burden and effort and increases in self-compassion and equanimity were expected (secondary outcomes). We further protocolled therapeutic techniques applied during the sessions and assumed a reduction in the total amount of such interventions across sessions. Concerning the neurobiological mechanisms, based on our prior work (Rosenbaum et al., 2021, Rosenbaum et al., 2020a, Rosenbaum et al., 2020b, Rosenbaum et al., 2018c), we hypothesized higher cortical activation in the CCN during training compared to control trials. This difference is expected to be reduced across and within sessions due to a learning effect: with ongoing training a shift from model-based (i.e. guided by the therapist) to model-free (i.e. internalized and more intuitive) ER should take place (Etkin et al., 2015). Finally, we expected a more pronounced CCN activity during the beginning compared to the middle and end of a session, due to a decline of prefrontal activity through CBT as shown for the treatment of anxiety disorders (Paquette et al., 2003, Rosenbaum et al., 2020b, Schrammen et al., 2022, Yang et al., 2014).

## 2 Materials and methods

### 2.1 Participants

We recruited participants via emails and flyers at the University Hospital of Tuebingen, the University of Tuebingen and via outpatient psychotherapists. All procedures are in line with the Declaration of Helsinki in its latest version and were approved by the ethics committee at the University Hospital and University of Tuebingen. The study protocol is registered at ClinicalTrials.gov (NCT04560192). All participants gave their written informed consent prior to data collection. Participants could be included in the study if they were between 18 and 60 years old, had never participated in a study with a Trier Social Stress Test (TSST) procedure and did not meet any of the following exclusion criteria: diabetes mellitus, kidney insufficiency, hypertension, dysrhythmia, cushing syndrome, current substance abuse, adrenal insufficiency, cortisone medication, pacemaker, craniocerebral trauma as well as any current primary mental or personality disorder, except ICD-10 diagnosis F32.x, F33.x and F34.1 in the group of the depressive sample (MDD). For this latter group, acute suicidality, extraordinarily severe depressive symptoms (BDI-II > 50) and strong decompensation under social stress in the past also led to exclusion. In the group of healthy controls (HC), only participants with low trait rumination (RRS score ≤ 2) and without any primary mental or personality disorder (current as well as in the past) were included.

**Fig. 1:**
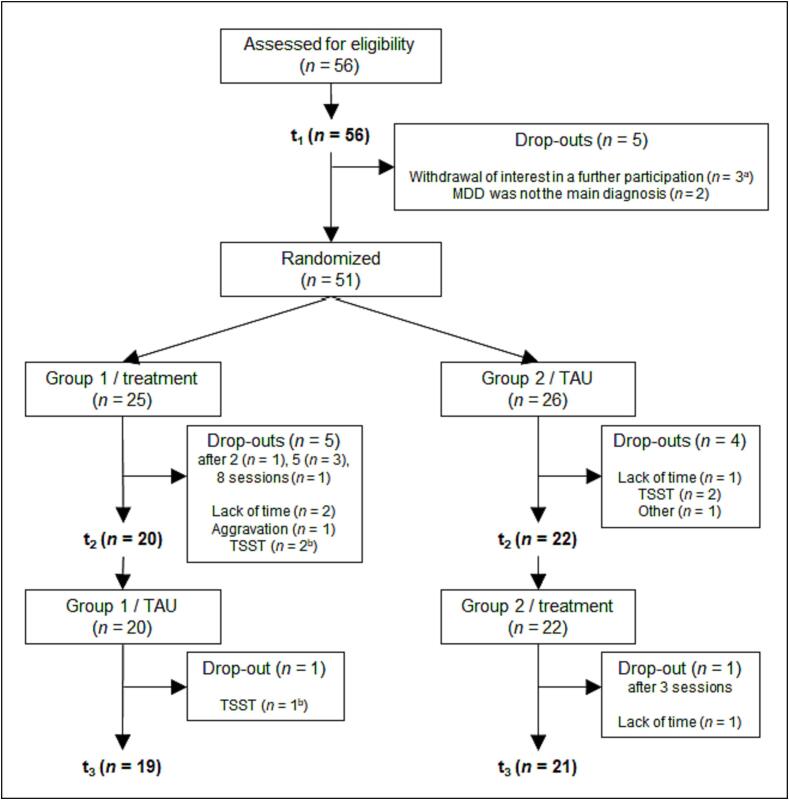
Flow of participants. *Note.* TSST = non-willingness to participate in another stress paradigm later in the study. ^a^One participant demanded all collected data to be deleted. ^b^Two patients did not complete the whole study procedure but participated in all eight therapeutic training sessions, so their data could be used in some of the conducted analyses.

In the final sample we collected data of 56 depressed patients and of 43 healthy controls. Sample sizes were determined by a previously performed power analysis that identified 42 patients and 42 healthy controls as needed. In the study at hand, only the clinical sample will be analyzed, which after dropouts was composed of the data of 42 patients (see Fig. 1). The mean age of this analyzed sample was 32.33 (*SD* = 10.98) years, and 69 % of the patients included in the analyses were female. The diagnoses in the analyzed patient sample included recurrent MDD (*n* = 40) and first episode MDD (*n* = 2). The average score in the BDI-II (Beck Depression Inventory II, Hautzinger et al., 2009) at the beginning of the study (t_1_) was 26.51 (*SD* = 7.78). Comorbid diagnoses included anxiety disorders (e.g. specific phobias such as acrophobia, social anxiety disorder; *n* = 16), eating disorders (in the past; *n* = 4), personality disorders (*n* = 2) and attention deficit hyperactivity disorder (*n* = 1). Twenty-two participants (52 %) had already had psychotherapeutic treatment(s) in their past. At the beginning of their study participation, 45 % were receiving psychotherapy and 45 % currently used antidepressant medication. In the course of the study participation, four participants started taking antidepressant medication and one began psychotherapy on an outpatient basis. On the other hand, two participants stopped their initial outpatient psychotherapy during study participation.

### 2.2 Procedures

**Fig. 2:**
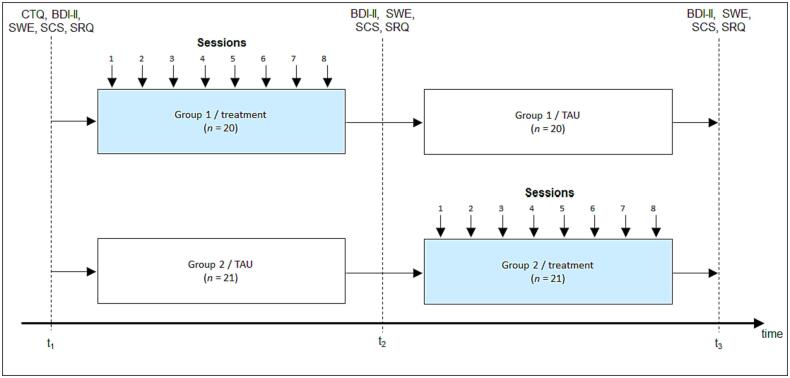
Study design of the project. Note. BDI-II = Beck-Depressions-Inventar II (Hautzinger et al., 2009). SWE = Skala zur Allgemeinen Selbstwirksamkeitserwartung (Jerusalem and Schwarzer, 2003). SCS = The Self-Compassion Scale (Hupfeld & Ruffieux, 2011). SRQ = state rumination questionnaire (see supplementary material). TAU = Treatment as usual. t_1_, t_2_, t_3_ = measurement points of the Trier Social Stress Test (TSST).

All measurements were performed in the premises of the Clinic for Psychiatry and Psychotherapy Tuebingen. For MDD patients a complete participation in the study lasted approximately ten weeks and consisted of twelve in-person appointments. All participants took part in a TSST (Kirschbaum et al., 1993) at the beginning (t_1_), the middle (t_2_) and the end (t_3_) of the study participation (a detailed description of the TSST can be found in the supplementals). The results of this stress test will be reported elsewhere. At t_1_ to t_3_, psychometric endpoints were assessed: depression symptom severity (BDI-II, Hautzinger et al., 2009), self-efficacy (Skala zur Allgemeinen Selbstwirksamkeitserwartung, SWE, Jerusalem and Schwarzer, 2003) and self-compassion (The Self-Compassion Scale German Version; SCS-D, Hupfeld & Ruffieux, 2011). Further, as part of the TSST ruminative thoughts were assessed three times during each TSST via a state rumination questionnaire (SRQ) that has been used in our previous studies (Rosenbaum et al., 2017, Rosenbaum et al., 2018b, Rosenbaum et al., 2018c, Rosenbaum et al., 2018d, Rosenbaum et al., 2020a; for items and scale statistics see supplementary material). To investigate overall changes associated with MBERT, we averaged state rumination measurements during each TSST and analyzed differences in rumination from t_1_ to t_3_ within and between the treatment groups. Further, as previous studies indicated strong associations between rumination and posttraumatic stress disorder (e.g. Arditte Hall et al., 2019, Michael et al., 2007), we assessed the German version of the Childhood Trauma Questionnaire (CTQ, Klinitzke et al., 2012) at the beginning of t_1_. MDD patients were block-randomized after completion of t_1_ (counterbalanced for sex and severity of depressive symptoms using Excel (Microsoft Corporation, 2018), depending on the corresponding values determined at t_1_) to either MBERT (*n* = 21) or TAU (*n* = 21). Initially (at t_1_), the groups did not differ in symptom severity, self-efficacy and rumination (BDI-II: *t*(39) = 0.811, *p_corr_* > 0.05, *d* = 0.25; SWE: *t*(39) = -0.963, *p_corr_* > 0.05, *d* = -0.30; SRQ: *t*(39) = -0.469, *p_corr_* > 0.05, *d* = -0.15), but in self-compassion (SCS: *t*(39) = 3.087, *p_corr_* < 0.05, *d* = 0.97), with significantly lower scores in the TAU group (see also Fig. 4a-d). For approximately four to five weeks, the treatment group received the MBERT consisting of one psychoeducative session and eight psychotherapeutic training sessions. After this first phase of the study, all patients completed a TSST (t_2_) before treatment and TAU groups switched. After completion of this phase a final TSST (t_3_) was assessed. Staff performing these TSSTs were blinded to the group assignment of the patients. We used this cross-over design to allow the combined analysis of the in-session fNIRS data of both study groups (see Fig. 2). Over the course of the whole study participation, patients were asked to answer ecological momentary assessments (EMA) about their current stress level, stressful events, rumination, equanimity, self-compassion, mindfulness and sleep quality twice per day. The results of the EMA data will be reported elsewhere.

#### 2.2.1 MBERT

**Fig. 3:**
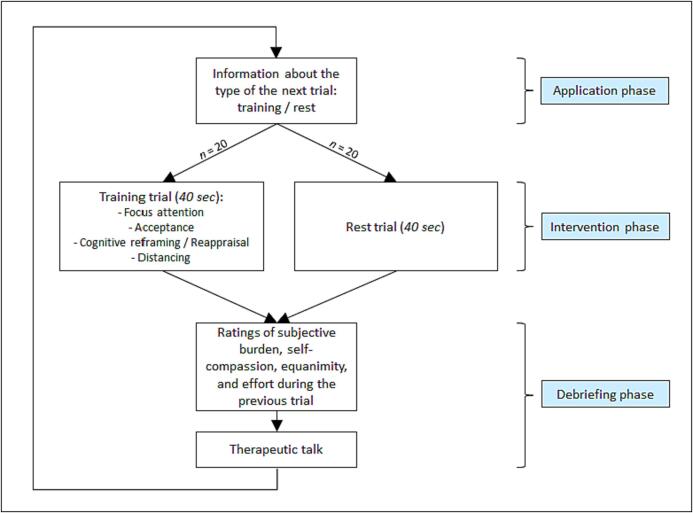
Procedure of the trials in the MBERT.

The psychotherapeutic training sessions followed a predefined script (see also Fig. 3) and were all executed by the same male psychotherapist in training (first author HL), who was supervised and in cases of illness represented by a senior CBT therapist (last author DR). The training started with a psychoeducative CBT session (50 min) on emotions, aberrant ER and how unresolved conflicts with personal goals, beliefs (schema) and needs in the long run can lead to rumination and depressive symptoms (Nolen-Hoeksema, 1991, O'Connor et al., 2007). The MBERT psychoeducative rumination model was based on a cognitive behavioral stimulus–response model, incorporating personal schemata, that conceptualized rumination as a secondary cognitive reaction to an unresolved emotional conflict. Based on this model, we conceptualized rumination not as a maladaptive ER strategy, but as a byproduct of absent adaptive ER that in itself causes prolonged stress and further negative consequences. The patients also got worksheets on the topic, including the identification of personal inner emotional conflicts as a first homework (see supplementary material: worksheets psychoeducation). Based on the psychoeducative concept, the mindfulness-based ER strategies, that were trained in each of the eight following training sessions, were explained: The patients were asked to think of (current) personal conflicts or themes they were ruminating about. In each session, the MBERT strategies were trained on one of those idiosyncratic topics. If necessary, the same conflict could be discussed in several sessions, which was the case nine times in total. After identifying the underlying primary emotion(s), the patients were instructed (1) to concentrate (strategy focus attention) on this affective state (respecting the whole emotional network including thoughts, action impulses, the focus of attention and the physical reaction), (2) to accept and tolerate the existence of these emotion(s) (considering the personal situation and experiences) in a mindful way (strategy acceptance), (3) to give them a new interpretation so the patients could look at them with a more even-minded view (strategy cognitive reframing / reappraisal), and (4) to cognitively and emotionally distance themselves from the negative conflict and the underlying negative emotion(s) (strategy distancing) (see Fig. 3). Please note that we selected and adapted those strategies from the manual of the relatively newly developed eclectic Emotion Regulation Therapy (Mennin et al., 2018, Mennin et al., 2015). In the eight psychotherapeutic training sessions, these four steps were gradually instructed and trained throughout 20 trials (à 40 s) in each session. Furthermore, these 20 training trials alternated in a semi-randomized manner with 20 control trials (à 40 s) in which patients were asked to rest and not to think about anything in particular. The randomization of the trials was block-randomized for every 10 trials (5 control and 5 training; i.e. after 10, 20, 30 and 40 trials). Each trial consisted of (1) a therapeutic talk / intervention (if needed), (2) an instruction by the therapist what kind of trial would follow and, in case of a training trial, what step of the strategy the patient should try to fulfill, (3) the trial of 40 s duration, and (4) a rating of the subjective burden, self-compassion, equanimity during the trial as well a rating of the effort to apply the strategy. In the therapeutic talk / intervention, helpful strategies to overcome problems with the ER strategies and instructions or assistance on the next trial were given. These instructions were composed of input specific to the MBERT strategies and general psychotherapeutic techniques such as validation, self-instructions, socratic questioning, metaphors, self-compassion, “pretending to”, chaining, motivational interviewing and control of body function. The used therapeutic interventions and techniques were noted for the corresponding trials in short on a session protocol by the psychologist.

During each training session, which took approximately 1 to 1.5 h, patients sat in front of a table on a comfortable chair with the fNIRS-cap on their head and the fNIRS-machine in their back. The psychotherapist was sitting on their left-hand side at an angle of approximately 90 degrees. As the study was conducted during the COVID-19 pandemic, for reasons of infection protection there was a plexiglass shield between the patients and the therapist and the latter wore a face mask.

Between sessions the patients were encouraged to practice the taught ER strategies in their daily lives and to also do mindfulness training and meditation using a free-access application (“Meditation Time”). For this purpose, they were further given worksheets they could use to go through the strategy of the sessions by themselves (see supplementary material: worksheets ER strategy 1 and 2). The therapeutic training sessions were delivered with two sessions per week, whereby there was at least one day of rest between two training sessions.

#### 2.2.2 fNIRS

During all therapeutic training sessions, an fNIRS measurement was performed to assess cortical oxygenated (O_2_Hb) and deoxygenated (HHb) blood using a continuous wave, multichannel NIRS system (ETG-4000 Optical Topography System; Hitachi Medical Co., Japan) with a temporal resolution of 10 Hz. For data recording a semiconductor laser and avalanche diodes at two wavelengths (695 ± 20 and 830 ± 20 nm) with 4.0 ± 0.2 mW for each wavelength at each optode were used. According to our regions of interest (ROIs), we placed two frontal probesets (with reference positions F3 and F4) and one parietal probeset (with reference positions Pz, P3, and P4), using an Easycap oriented on Fpz and Cz according to the 10–20 system (Jasper, 1958) with sponge rings for additional fixation of the optodes (see Table 1 and supplementary material Figure S1) and with a fixed inter-optode distance of 3 cm.

**Table 1:** Regions of interest and corresponding probesets and channels, extrapolated based on the Colin 27 template (Cutini et al., 2011). For probeset placement see also Figure S1.

region of interest	probeset	corresponding channels
left inferior frontal gyrus (lIFG)	left frontal	6 7 9
left dorsolateral prefrontal cortex (lDLPFC)	left frontal	10 11 12
right inferior frontal gyrus (rIFG)	right frontal	18 19 21
right dorsolateral prefrontal cortex (rDLPFC)	right frontal	20 23 24
somatosensory association cortex (SAC)	parietal	25 26 27 28 30 31 32 35 36

After export of the NIRS data, we computed changes in O_2_Hb and HHb by means of a modified Beer-Lambert law. Data preprocessing was performed with MATLAB 2020a (MathWorks Inc, 2020) using customized scripts, including interpolation of single noisy channels, correction of motion artifacts using Temporal Derivative Distribution Repair (TDDR) in order to remove spikes primarily caused by movements (Fishburn et al., 2019) and Correlation-based signal improvement (CBSI) (Cui et al., 2010). Further, through the cbsi-algorithm, the signals of O_2_Hb (high sensitivity) and HHb (high resilience to arousal artifacts) were included in one signal of corrected O_2_Hb, which is why only this data was used for analysis. We further used bandpass-filtering to remove low-frequency baseline-drifts (below 0.01 Hz) and high-frequency noise (above 0.1 Hz). Then, a second step of channel interpolation followed in case of artifacts due to data correction and further single artifact-loaded channels were interpolated after visual inspection. Afterwards, a global signal reduction was performed with a spatial Gaussian kernel filter with a standard deviation of *σ* = 40 (Zhang et al., 2016) before z-transforming the data for comparison between subjects. Event-related averages were computed over the first, second and third 33 % of the sessions for training and control trials separately. Data was averaged in 5 ROIs comprising the somatosensory association cortex (SAC), bilateral DLPFC and IFG (see Table 1). ROIs were selected according to previous studies on the CCN (Rosenbaum et al., 2018b, Rosenbaum et al., 2018c, Rosenbaum et al., 2018d).

### 2.3 Data analysis

To analyze the overall efficacy of the MBERT, we analyzed changes in depressive symptoms, self-efficacy, self-compassion and state rumination by using the data of corresponding questionnaires (BDI-II, SWE, SCS, SRQ). We conducted a repeated measurements multivariate analysis of variance (rmMANOVA) for the factor group (treatment vs. TAU) and measurement point (t_1_ vs. t_2_ vs. t_3_) on these questionnaire data. Post-hoc comparisons within (t_1_ vs. t_3_) and between the two groups (at each of the time points) were done using pairwise as well as independent t-tests, corrected for multiple testing by the Benjamini-Hochberg procedure (in total twelve post-hoc comparisons were calculated). Within and between-session changes in the subjective ratings of subjective burden, self-compassion, equanimity and effort as well as fNIRS data were also assessed using rmMANOVAs. For reasons of simplicity, we directly computed the contrast (i.e. the difference) between the training and the control trials. Concerning the subjective ratings, we included the factors session (first to eighth) and session phase (beginning vs. middle vs. end of session). Hypotheses regarding the fNIRS data were investigated using the factors session (first to eighth), session phase (first vs. middle vs. end of session) and as dependent variables O_2_Hb levels of the different ROIs (bilateral DLPFC, bilateral IFG, SAC). For all rmMANOVAs polynomial contrasts were used for post-hoc tests as we hypothesized linear changes in the questionnaire data, the subjective ratings as well as in hemodynamic responses during the sessions. Note that we will not report polynomial contrasts higher than cubic order as they resulted most likely due to spurious fluctuations. If sphericity was violated, we used the Huynh-Feldt correction (Huynh & Feldt, 1976). All post-hoc comparisons were corrected using the procedure of Benjamini-Hochberg and adjusted p-values are reported (*p*_*corr*_) (Chen et al., 2017). Note that we further performed additional exploratory analyses for all of these three rmMANOVAs respecting (a) childhood traumatization (CTQ), (b) sex and (c) medication as categorical factors, which might possibly influence our results on a behavioral as well as on a neural level. However, these analyses were all non-significant. For reasons of a simpler illustration of our results concerning our main hypotheses, we refrained from presenting the results of these additional analyses.

We analyzed the used therapeutic techniques that have been instructed during the therapeutic talk phase. This qualitative data was categorized into 11 interventions: Attention regulation and focus (e.g. on the main emotion in a burdening topic and its emotional network), chaining (e.g. linking different aspects or substeps of the used strategies), cognitive perspective change (consisting of acceptance (e.g. of negative emotions), cognitive reframing / reappraisal (e.g. seeing the functional aspects of negative emotions, such as signals of needs) and self-compassion (e.g. giving oneself the same kindness and care as if it was to a good friend)), distancing and equanimity (e.g. observing an actual burdening emotion from a distanced perspective), metaphor (e.g. seeing anxiety as a smoke detector / alarm signal), motivational interviewing, self-verbalization / self-instruction (e.g. formulation of self-verbalization “Even if I feel weak sometimes, I’m not a failure”), socratic questioning, “pretending to” (e.g. thinking about how a situation would be without a certain negative emotion), validation (e.g. seeing that every person would have certain emotions in situations the patient is burdened by) and control of body function (e.g. controlled breathing or relaxation). Using Chi-squared tests we analyzed if the frequency of intervention implementation of any as well as of specific interventions differed between sessions and session phases.

We finally conducted an exploratory analysis to investigate the relationship between behavioral ratings and O_2_Hb levels. Using linear mixed models, we modeled the subjective ratings as dependent variables from session, session phase, previous subjective ratings as a lagged variable (for the investigation of changes in ratings) and O_2_Hb level, separated into a between- (BP_O_2_Hb) and a within-person factor (WP_O_2_Hb) by person mean centering. Random intercepts were modeled and the O_2_Hb levels were used as continuous covariates.

All analyses were done using R (R Core Team, 2020) and SPSS (Corp, 2020). In R the packages lme4 (Bates et al., 2014) and lmerTEST (Kuznetsova et al., 2017) were used for fitting mixed models to obtain *p*-values using the Satterthwaite approximation. Further, the R-package MuMIn (Barton, 2020) was used for calculating marginal *R*^2^ as a measure of variance explained by the fixed effects in the mixed models. Graphics were plotted using the R-package ggplot2 (Wickham, 2009).

## 3 Results

### 3.1 Efficacy compared to waiting list: Questionnaires

Data of 41 patients could be included in the analyses of the questionnaire data (see Fig. 1). For descriptive statistics of these data, separated by groups, see Table S5.

**Fig. 4:**
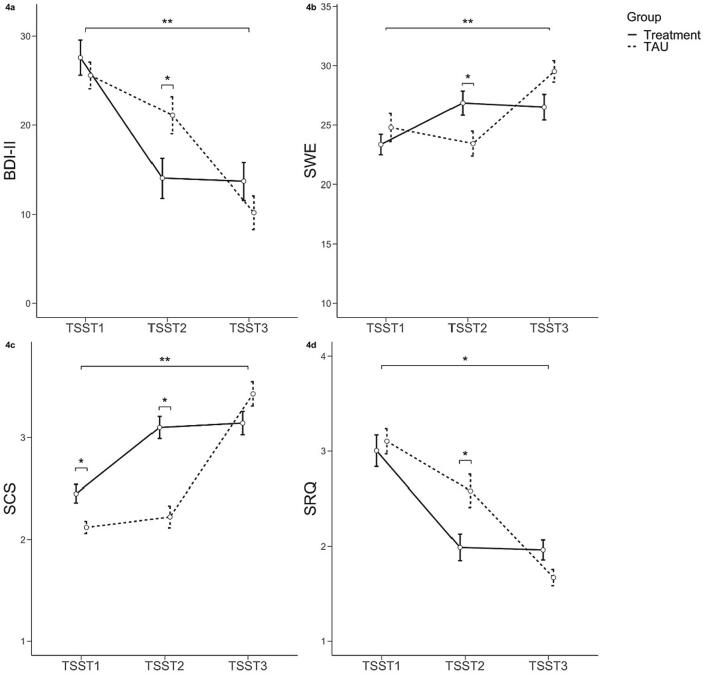
Changes in depressive symptom severity (4a), self-efficacy (4b), self-compassion (4c) and ruminative thoughts (4d) in the course of the study participation, differentiated by group (treatment vs. TAU). Small brackets symbolize significant group differences.

Our analyses revealed a significant interaction effect for time and group (*Wilks λ* = 0.511, *F*(8,146) = 7.268, *p* <.001, *ηp^2^* = 0.29), composed of significant interactions for all of the questionnaires (BDI-II: *F*(2,76) = 7.351, *p_corr_* < 0.05, *ηp^2^* = 0.16; SWE: *F*(2,76) = 14.305, *p_corr_* < 0.05, *ηp^2^* = 0.27; SCS: *F*(2,76) = 28.856, *p_corr_* < 0.05, *ηp^2^* = 0.43; SRQ: *F*(2,76) = 6.986, *p_corr_* < 0.05, *ηp^2^* = 0.16). All of these interactions were characterized by a quadratic relationship (BDI-II: *F*(1,38) = 17.687, *p* <.001, *ηp^2^* = 0.32; SWE: *F*(1,38) = 38.818, *p* <.001, *ηp^2^* = 0.51; SCS: *F*(1,38) = 40.492, *p* <.001, *ηp^2^* = 0.52; SRQ: *F*(1,38) = 11.353, *p* <.01, *ηp^2^* = 0.23), indicating expected time delayed u-shaped changes between the groups (see Fig. 4a-d). Post-hoc comparisons from the beginning (t_1_) to the end of the study (t_3_) revealed high effect sizes in all measures (BDI-II: *t*(40) = -9.411, *p_corr_* < 0.01, *d* = -1.47; SWE: *t*(40) = 5.611, *p_corr_* < 0.01, *d* = 0.88; SCS: *t*(40) = 11.170, *p_corr_* < 0.01, *d* = 1.75; SRQ: *t*(40) = -11.688, *p_corr_* < 0.05, *d* = -1.85). At t_2_ the between-group differences were significant in all measures (BDI-II: *t*(39) = -2.330, *p_corr_* < 0.05, *d* = -0.73; SWE: *t*(39) = 2.346, *p_corr_* < 0.05, *d* = 0.73; SCS: *t*(39) = 5.768, *p_corr_* < 0.05, *d* = 1.80; SRQ: *t*(39) = -2.632, *p_corr_* < 0.05, *d* = -0.82), indicating a stronger reduction in symptom severity and rumination and a stronger increase in self-efficacy and self-compassion due to MBERT. Additionally, we checked if groups differed at t_3_, which was not the case for any of the four measures (BDI-II: *t*(39) = 1.232, *p_corr_* > 0.05, *d* = 0.39; SWE: *t*(39) = -2.169, *p_corr_* > 0.05, *d* = -0.68; SCS: *t*(39) = -1.736, *p_corr_* > 0.05, *d* = -0.54; SRQ: *t*(39) = 2.155, *p_corr_* > 0.05, *d* = 0.68) (see Fig. 4a-d).

### 3.2 Therapeutic techniques

In the application of additional psychotherapeutic interventions during the talking phase between trials, we found a significant decrease in intervention use from session to session (session 1 = 14 %, session 2 = 13.8 %, session 3 = 12.5 %, session 4 = 12.4 %, session 5 = 12.5 %, session 6 = 11.8 %, session 7 = 11.5 %, session 8 = 11.4 %; *χ^2^*(7) = 20.675, *p* <.01) and that intervention use within the sessions decreased significantly over the session phases with most of the interventions being performed during the first and the second third (first third = 39.2 %, second third = 42.4 %, last third = 18.3 %; *χ^2^*(2) = 357.465, *p* <.001). Further, the comparison of the applied interventions revealed significant differences in the frequency of their particular realization (*χ^2^*(12) = 2621.511, *p* <.001) as well as within different session phases (*χ^2^*(24) = 2902.915, *p* <.001), but not different sessions (*χ^2^*(84) = 84.757, *p* >.1).

Overall, additional instructions on cognitive perspective change (cognitive reframing / reappraisal (22.2 %), acceptance (10.6 %) and self-compassion (13.4 %)) were used most frequently (46.2 %), followed by validation (15.6 %), distancing and equanimity (14.4 %) and attention regulation (13.6 %). All other interventions (metaphors, motivation, self-instruction, control of body function, socratic questioning, chaining, pretending to; sorted in descending order) were used less than 5 %, respectively (see Fig. 5a). Further, attention regulation, validation as well as acceptance were used predominantly at the beginning of the sessions before a new dealing with or an alternative perspective on the problem / topic could be installed. For this latter purpose, cognitive reframing / reappraisal and self-compassion (as well as all the less frequently used strategies) were used mostly in the middle of the sessions, whereas distancing and equanimity particularly served as closure of the sessions (see Fig. 5b). Investigated separately, instructions on acceptance, attention regulation, validation as well as cognitive reframing / reappraisal decreased from the first to the last session. Self-compassion as well as distancing and equanimity were used consistently over sessions. However, throughout the sessions instructions on distancing and equanimity increased in the first and the second phase and decreased in the last third of the session (see Fig. 5b).

**Fig. 5a:**
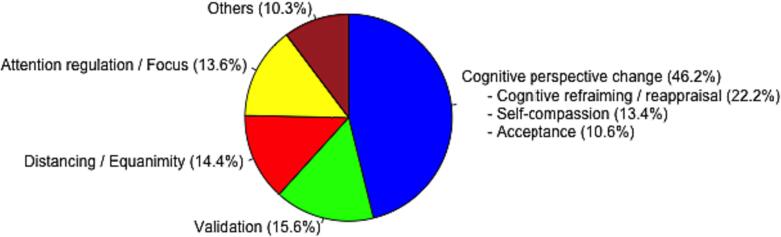
Overall amount of given therapeutic interventions.

**Fig. 5b:**
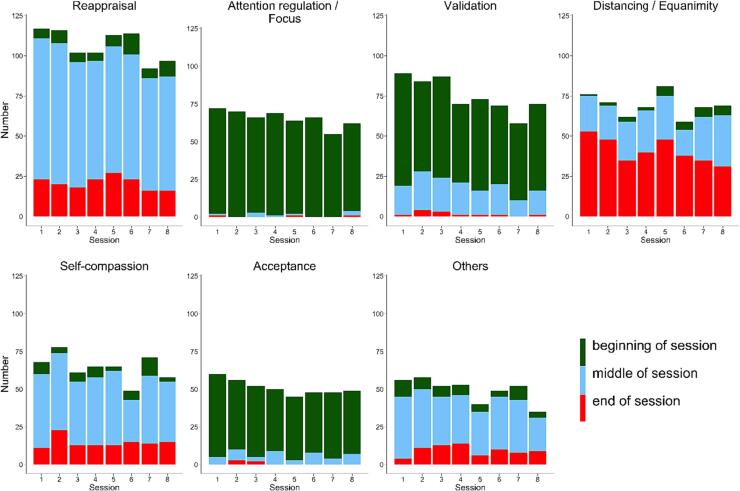
Amount of therapeutic interventions/instructions, differentiated by sessions and session phases.

*Note.* Others contain: metaphors, motivation, self-instruction, control of body function, socratic questioning, chaining, pretending to.

### 3.3 Subjective ratings

The results concerning the subjective ratings are based on the experimental contrast (training-control). We found a highly significant constant term, representing a main effect of the condition contrast (*Wilks λ* = 0.293, *F*(4,38) = 22.914, *p* <.001, *ηp^2^* = 0.71) in general as well as for each of the four subjective ratings (subjective burden: *F*(1,41) = 75.531, *p* <.001, *ηp^2^* = 0.65; self-compassion: *F*(1,41) = 11.211, *p* <.01, *ηp^2^* = 0.22*;* equanimity: *F*(1,41) = 59.198, *p* <.001, *ηp^2^* = 0.59; effort: *F*(1,41) = 45.203, *p* <.001, *ηp^2^* = 0.51), representing higher subjective burden and effort as well as lower equanimity and self-compassion during training compared to control trials. Further, our rmMANOVA revealed main effects for session phase (*Wilks λ* = 0.33, *F*(8,158) = 14.641, *p* <.001, *ηp^2^* = 0.43) as well as for session (*Wilks λ* = 0.858, *F*(28,1025.399) = 1.594, *p* <.05, *ηp^2^* = 0.04).

**Fig. 6:**
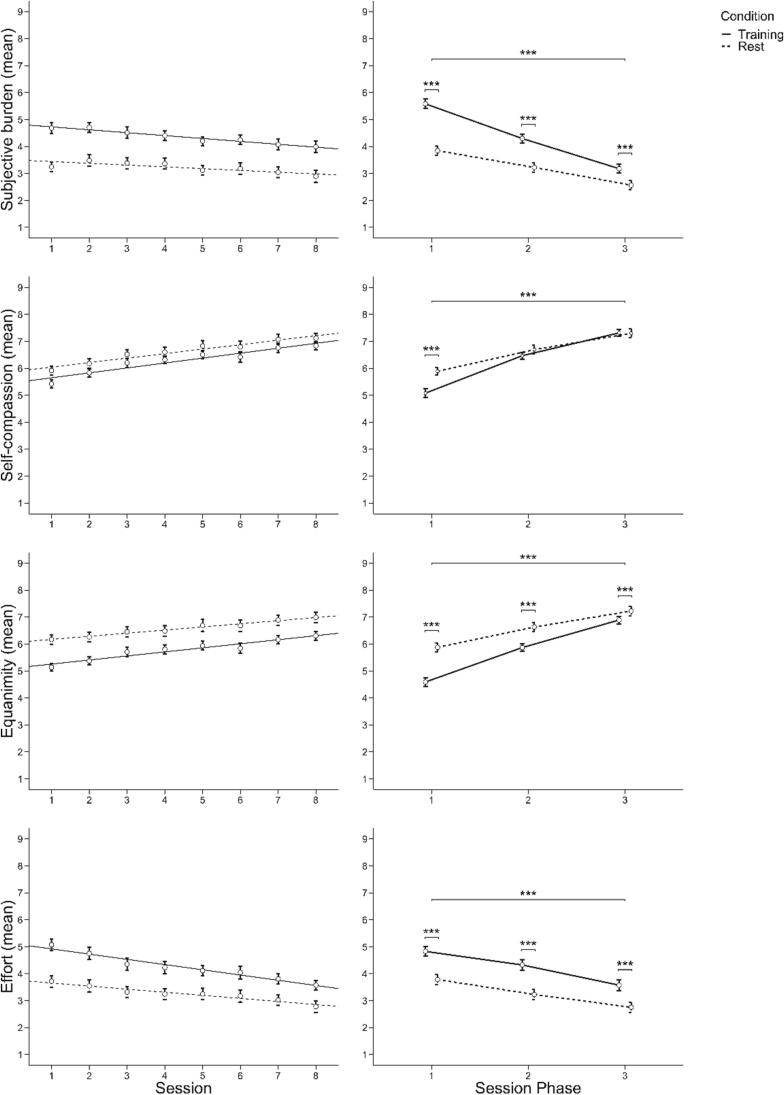
Changes in subjective rating means over sessions and session phases.

With respect to the main effect of session phase, all contrasts changed significantly within the three session phases with a decrease in the case of subjective burden (Huynh-Feldt *F*(2,82) = 60.795, *p* <.001, *ηp^2^* = 0.60) and effort (Huynh-Feldt *F*(2,82) = 4.065, *p* <.05, *ηp^2^* = 0.09) and an increase in the case of self-compassion (Huynh-Feldt *F*(2,82) = 53.443, *p* <.001, *ηp^2^* = 0.57) and equanimity (Huynh-Feldt: *F*(2,82) = 58.529, *p* <.001, *ηp^2^* = 0.59). These changes were characterized by a linear relationship in the case of subjective burden (*F*(1,41) = 73.285, *p* <.001, *ηp^2^* = 0.64), self-compassion (*F*(1,41) = 61.035, *p* <.001, *ηp^2^* = 0.60) and equanimity (*F*(1,41) = 70.081, *p* <.001, *ηp^2^* = 0.63), indicating constant changes over the session phases. Note that these changes of the condition contrasts were caused by higher changes in the subjective ratings in training compared to control trials, i.e. higher reductions in subjective burden and higher increases in self-compassion and equanimity during training than control trials. For the subjective rating of effort as well as of self-compassion, the found main effects were further characterized also by quadratic relationships (self-compassion: *F*(1,41) = 17.165, *p* <.001, *ηp^2^* = 0.3; effort: *F*(1,41) = 9.230, *p* <.01, *ηp^2^* = 0.18), reflecting a stronger increase from the first to the second and a smaller one from the second to the third session phase in the case of self-compassion and a small increase followed by a strong decrease of the condition contrast in the case of effort (see Fig. 6).

Concerning the main effect of session, the condition contrasts of subjective burden and effort decreased significantly (subjective burden: Huynh-Feldt *F*(7,287) = 3.050, *p_corr_* < 0.05, *ηp^2^* = 0.07; effort: Huynh-Feldt *F*(7,287) = 3.887, *p_corr_* < 0.05, *ηp^2^* = 0.09), whereas equanimity increased significantly over sessions (Huynh-Feldt *F*(7,287) = 2.501, *p_corr_* < 0.05, *ηp^2^* = 0.06). All of these changes were characterized by a linear relationship (subjective burden: *F*(1,41) = 10.598, *p* <.01, *ηp^2^* = 0.21; equanimity: *F*(1,41) = 4.750, *p* <.05, *ηp^2^* = 0.1; effort: *F*(1,41) = 12.558, *p* <.01, *ηp^2^* = 0.23), reflecting constant changes over the sessions with decreases in subjective burden and effort and increases in equanimity. Further, the main effect of subjective burden was also characterized by a quadratic relationship (*F*(1,41) = 7.271, *p* <.05, *ηp^2^* = 0.15), indicating a reduction in the first sessions and a small resurgence of the condition contrast in the final sessions (see Fig. 6).

Further, no significant interaction of session and session phase was observed.

### 3.4 fNIRS data

All fNIRS results are based on the experimental contrast describing the difference of the therapeutic training trials and the control trials. Due to bad data quality, the data of five training sessions was imputed via multiple imputation with five iterations before running the analyses. Please note that inclusion and exclusion of the related patients did not lead to changes in the results.

**Fig. 7:**
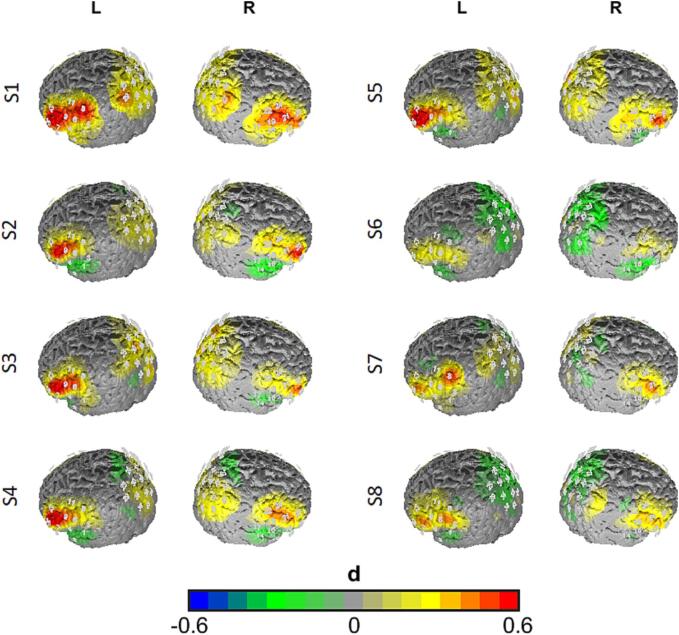
Activation maps for the experimental contrast (training vs. rest) for the different sessions (S1 to S8; top to bottom). Differences are plotted as effect sizes in Cohen's d. Warm colors indicate higher activation during training trials, while cold colors indicate higher activation in rest trials.

We found a highly significant constant term, representing a main effect of the condition contrast in general (*Wilks λ* = 0.439, *F*(5,37) = 9.468, *p* <.001, *ηp^2^* = 0.56). Univariate analyses of this effect revealed significant effects in the bilateral IFG and the bilateral DLPFC (rIFG: *F*(1,41) = 21.830, *p* <.001, *ηp^2^* = 0.35; lIFG: *F*(1,41) = 35.790, *p* <.001, *ηp^2^* = 0.47; rDLPFC: *F*(1,41) = 14.320, *p* <.001, *ηp^2^* = 0.26; lDLPFC: *F*(1,41) = 15.869, *p* <.001, *ηp^2^* = 0.28), indicating higher O_2_Hb levels during training compared to control trials in the prefrontal cortex. Further, our analyses revealed a main effect for session (*Wilks λ* = 0.821, *F*(35,1192.903) = 1.636, *p* <.05, *ηp^2^* = 0.04). More precisely, the condition contrast of O_2_Hb levels decreased from session to session in the bilateral DLPFC (lDLPFC: *F*(7,287) = 3.501, *p_corr_* < 0.05, *ηp^2^* = 0.08; rDLPFC: Huynh-Feldt *F*(7,287) = 3.516, *p_corr_* < 0.05, *ηp^2^* = 0.08). Both main effects were characterized by a linear relationship (lDLPFC: *F*(1,41) = 12.168, *p* <.01, *ηp^2^* = 0.23; rDLPFC: *F*(1,41) = 10.392, *p* <.01, *ηp^2^* = 0.2), indicating a constant reduction over the sessions in these ROIs (see Fig. 7).

### 3.5 Exploratory analysis

Finally, we analyzed the association of the subjective ratings with the time variables session and session phase as well as with cortical oxygenation by fitting mixed models, separately for each ROI and each subjective rating. Note that we included only training trials in this analysis to reduce the complexity of the models. The results are to be found in Table 2. As expected from the previous analysis, we observed significant main effects for session and session phase and an interaction of session by phase for all dependent variables except for subjective burden. Concerning the associations with cortical oxygenation, we found a significant main effect for the within-person predictor of the O_2_Hb variable in the SAC for all subjective ratings. More precisely, our results showed significant positive associations of the individual fluctuation in cortical oxygenation in the SAC with self-compassion as well as with equanimity and negative associations with subjective burden and effort. Moreover, for the rating of effort the negative association was also significant in the bilateral DLPFC and IFG. Interestingly, we also observed a positive interaction of within-person O_2_Hb levels and the predictors of session and session phase, indicating that within each session and from session to session, increased cortical oxygenation within prefrontal areas was positively associated with subjective effort. Furthermore, the positive association of session and O_2_Hb levels was also found for between-person levels of cortical oxygenation in the SAC for the ratings of subjective burden, indicating that with increasing sessions patients showing generally higher cortical oxygenation showed higher subjective burden. Similarly, we observed an interaction of session phase and between-subject O_2_Hb levels in the IFG for the ratings of subjective burden, self-compassion and equanimity. With longer durations of the session, IFG activation was positively associated with subjective burden and negatively associated with self-compassion and equanimity between subjects, i.e. patients with higher IFG activation showed higher effort, lower self-compassion and lower equanimity than subjects with lower IFG activation in later phases of the session.

**Table 2:** Results of the mixed models exploring the association between the subjective ratings, cortical oxygenation in the different ROIs, sessions and session phases in the training trials(Session:Phase:WP_O_2_Hb + Session:Phase:BP_O_2_Hb + Rating_lagged).

Beta-estimates and standard errors in brackets. AIC = Akaike Information Criterion; BIC = Bayesian-Information-Criterion; R^2^ = variance explained by the fixed effects. Significant results are shaded whereby darker shadows indicate smaller *p*-values. #*p* <.1, *p <.05, **p <.01, ***p <.001.

Please note that we also performed the analysis with no auto-regressor, as well as with non-centered predictors which yielded the same results (see supplementary material Tables S6 and S7).

## 4 Discussion

The purpose of this study was to investigate the efficacy and neural correlates of a MBERT in the treatment of depressive rumination. More specifically, we aimed to shed light on the neural mechanisms related to typical trained emotion regulation skills in CBT. To this end, 42 subjects suffering from MDD completed an eight-session treatment in which ER strategies were taught and practiced, while cortical blood oxygenation was assessed with fNIRS. We examined the efficacy of the training on a behavioral level using psychometric measures. Impacts of the training on a psychological and neural level were investigated using subjective ratings on session variables and hemodynamic responses during the training in the CCN. We found significant and treatment-specific changes in depressive symptom severity, self-compassion, self-efficacy and ruminative thoughts after completion of the MBERT in comparison to TAU. During and between training sessions, subjective ratings on burden, self-compassion, equanimity and effort changed significantly in expected change patterns, i.e. increases in self-compassion and equanimity and decreases in subjective burden and effort. The amount of implemented therapeutic techniques provided by the therapist between the trials of the training decreased during as well as between sessions, indicating that less therapeutic guidance was needed with ongoing treatment. Concerning the neural correlates, we found higher cortical activation during training compared to rest trials in the prefrontal areas of the CCN, namely in the bilateral DLPFC and the bilateral IFG. As hypothesized, this difference between conditions decreased significantly across sessions in the bilateral DLPFC with a linear decrease. However, no significant reduction of the condition contrast was found within each session. Finally, an exploratory analysis indicated associations of the individual cortical oxygenation in the CCN and process-related subjective ratings during the training. Individual fluctuations of CCN oxygenation above the individual mean at the beginning of the training was associated with reduced subjective effort and burden, as well as increased self-compassion and equanimity. However, with procession within each training session and between training sessions, remaining higher intra- und inter-subject levels of O_2_Hb in the CCN were associated in the inverse direction, i.e. increased effort and subjective burden, as well as decreased self-compassion and equanimity.

As the designed MBERT training is composed of typical CBT components, it is not surprising that the observed efficacy is well in line with previous studies, showing that CBT is an effective treatment of MDD (Beck and Alford, 2014, Papageorgiou and Wells, 2004, Renna et al., 2017). Our results emphasize that mindfulness-based emotion regulation (Baer, 2003, Deyo et al., 2009, Hofmann et al., 2010, Jain et al., 2007, Laicher et al., 2022, Mennin et al., 2015) is an effective component in treating rumination in MDD (Watkins et al., 2011, Wells and Matthews, 1996, Wells and Matthews, 2014, Wells et al., 2011). In our study, relatively short periods of intensive ER training resulted in highly significant reductions in symptom severity and ruminative thoughts, increases in self-compassion and self-efficacy compared to TAU. The efficacy is further supported by in-session measurements of subjective burden, self-compassion, equanimity and effort that showed constant improvements within and across sessions with slightly faster improvements in the first compared to the last sessions. This is well in line with previous findings that the strongest symptomatic changes seem to take place in the first sessions and weeks of psychotherapy (Howard et al., 1986, Leder and Porter, 1968). Interestingly, this initial improvement coincides with the frequency of therapeutic interventions given, which also became less within and across sessions. The reduction in therapeutic guidance is coherent with the general rationale of CBT that clients are “taught over time to serve as their own therapists, to apply the principles of CBT with decreasing amounts of guidance from the therapist” (p.452, Roth et al., 2002). Nevertheless, significant symptom reduction persisted even with less therapeutic guidance at the end of each session as well as in the later sessions in general. On a neural level, activation was significantly higher during the training than during rest trials in the CCN, especially in the bilateral IFG and the bilateral DLPFC. This contrast was attenuated in the first session and decreased from session to session. Increased cortical activity in the CCN was expected as the functions of the CCN, e.g. attention regulation, working memory, executive control and inhibition, are required in the ER strategies learned during MBERT. First, attentional deployment towards the burdening topic was required, followed by active focus and keeping the topic and the corresponding (negative) emotions in mind, before using other cognitive ER strategies such as perspective change (e.g. acceptance and reframing). All of those tasks are known to be associated with activity in the CCN (Cole and Schneider, 2007, Rosenbaum et al., 2016, Rossi et al., 2009). Interestingly, the observed reduction of O_2_Hb levels over sessions is well in line with previous findings of our group in exposure therapy for arachnophobia (Rosenbaum et al., 2020b). In this study, we also observed higher CCN activity in the beginning of the sessions and the first sessions that decreased over the course of therapy. This could be explained by theoretical accounts of model-based and model-free emotion regulation (Etkin et al., 2015). At the beginning of the training, model-based (guided by plans) ER was predominant. With the progression of the training, patients got familiar with the trained ER strategies which might have caused a lower need of cognitive activation to fulfill the requested steps, as routine learning took place. Correspondingly, previous investigations found reductions in prefrontal cortical activity due to (cognitive behavioral) therapy (Brody et al., 2001, Goldapple et al., 2004, Marwood et al., 2018, Rosenbaum et al., 2020b, Schrammen et al., 2022). This interpretation is further supported by our exploratory analysis. In general, subjective burden and CCN activity decreased over the course of treatment. However, effort and subjective burden were increasingly positively associated with CCN activity during later phases of the sessions and with progression of the training. This might be explained by patients that are struggling with the implementation of the ER strategies and the change from model-based to model-free ER strategies: their effort and CCN activity might have stayed higher compared to the adapted patients, which results in the more pronounced positive correlation at later sessions observed here. Contrary to our hypothesis, we did not observe reductions of the condition contrast within sessions, but only between sessions. From our point of view, the most likely explanation for this null-result is that in contrast to our previous study on exposure therapy, different ER strategies have been deployed and the change from model-based to model-free ER needed longer. In the treatment of “simple” phobias, the deployed ER strategies are rather non-complex, while the treatment of depression-related emotional conflicts often times involves complex schemata, e.g. based on experiences in different relationships. In line with this, we used more sessions in this study on depressed patients than in the treatment protocol for simple phobias. In future studies it might be possible to reduce the number of trained ER strategies – however, many aspects that we treated as separate strategies (e.g. attention regulation, acceptance, reframing, distancing) are in fact part of any “simple ER strategy”. For example, reframing is not possible without focusing one’s attention, acceptance can be seen as a special form of reframing or cognitive perspective change and all ER strategies involve "letting go” of the emotional subject at hand (i.e. distancing). In fact, those different strategies might be seen as one category “cognitive ER / cognitive perspective change” that has different facets that are merely differentiated on an academic level.

It is known from previous studies that there are differences in the epidemiology and the course of depression between sexes, both on a behavioral as well as a neural level (Culbertson, 1997, Mohammadi et al., 2023, Nolen-Hoeksema, 1990). Therefore, we performed additional analyses to investigate if our results were moderated by sex. Although this was not the case, it needs to be mentioned that the analyzed sample only included 13 male subjects. Additionally, as trauma-related experiences were also found to influence ruminative processes, we further performed analyses respecting childhood traumatization. As for the factor sex, we did not find any significant influence. However, our interpretation concerning the influence of traumatization on (depressive) rumination is limited to retrospective traumatic experiences from the childhood as subjects were only asked to answer the CTQ. Furthermore, since some patients were using antidepressant medication, we conducted analyses taking into consideration their medication intake. Once again, no significant influence was observed. However, it is important to keep in mind that we did not differentiate between types of antidepressant medication or consider dosage due to limited data.

Some limitations have to be addressed. First of all, even though the training was found to be highly effective, we did not collect follow-up data to examine the long-term stability of the effects. At least the effects obtained seemed to remain stable over five weeks, as we could observe by the cross-over design in those subjects that first received the training. To verify long-term effectiveness, comprehensive follow-up surveys on a larger sample and after several months would be needed. However, it was not the aim of this study to create a new treatment method as a standalone treatment, but to primarily shed light on the mechanisms and neural correlates of CBT-specific treatment components. Second, it cannot be ruled out that patients may have been influenced in their subjective ratings and the answering of the psychometric questionnaires by the presumed expectations of the investigators (Connor & Callahan, 2015). Due to restrictions in the financial resources of this project, it was not possible to include an active control-treatment, as this was the first project that combines CBT-specific treatment components and in situ fNIRS measurements in MDD. Future studies will be needed to address this point by including active control conditions. Furthermore, it must be kept in mind that the found effects cannot be separated into specific (e.g. technique) and nonspecific factors (e.g. therapeutic relationship) of the intervention. Again, comparative active treatment control conditions are needed. Given potential influences of interindividual differences in executive functions (e.g. working memory), it is important to acknowledge that the interpretation of our results regarding the effects of MBERT on subjective burden and effort may be limited. This limitation arises from the fact that MBERT itself can be considered cognitively demanding. Consequently, individuals with lower executive functions may have perceived greater subjective burden and effort during the training compared to those with higher executive functions. However, since we did not collect baseline data on the general executive functions of our subjects, we were unable to assess the potential influence of this factor. Finally, we used a non-active rest control condition for comparison to the training trials. In the design of the study we decided to use this non-active control condition to exclude “carry-over” effects from the control-condition towards the training trials. In one of our previous pilot studies, active control conditions such as thinking about the future induced CCN activity due to the mental process. Indeed, from our perspective it seems to be nearly impossible to create a sham ER strategies technique that could be used for the control condition without activation of the CCN. The design and development of more advanced control conditions for the investigation of specific psychotherapeutic techniques within the treatment context, as realized in this study, will be an important step for future investigations. In our study, we used fNIRS to investigate the neural correlates of psychotherapeutic techniques in an ecologically valid setting. Even though fNIRS is very useful due to its reduced susceptibility to motor artifacts (Ehlis et al., 2014, Hajime, 2013, Jue and Masuda, 2013), it is important to acknowledge that fNIRS has a limited penetration depth, reaching approximately 1.5–2 cm into the cortex (Haeussinger et al., 2011). Consequently, we were unable to investigate the effects of MBERT on other brain regions that seem to be additionally relevant in the context of depressive rumination, such as the areas of the DMN (Burkhouse et al., 2017, Hamilton et al., 2011). As our study emphasizes the role of the prefrontal cortex especially in the beginning of the treatment, it will be an interesting endeavor for future studies to combine neurostimulation techniques with ER trainings in these brain areas to enhance the efficacy of psychotherapeutic techniques.

## 5 Conclusion

Taken together, the study at hand showed that ER strategies that are typical components of CBT activate prefrontal areas of the CCN at the beginning of treatment. In line with theoretical accounts of model-based and model-free emotion regulation, the training of those skills results in decreases of activity in the CCN over time which is accompanied by reductions in subjective effort, burden and increases in self-compassion and equanimity.

## Funding

This work was supported by the fortune funding program at the University of Tuebingen [grant number F1331582]. We further acknowledge support from the Open Access Publication Fund of the University of Tuebingen.

## CRediT authorship contribution statement

**Hendrik Laicher:** Conceptualization, Methodology, Software, Formal analysis, Investigation, Resources, Data curation, Writing – original draft, Writing – review & editing, Visualization. **Isabell Int-Veen:** Investigation, Resources, Data curation, Writing – review & editing. **Leonie Woloszyn:** Investigation, Writing – review & editing. **Ariane Wiegand:** Writing – review & editing. **Agnes Kroczek:** Writing – review & editing. **Daniel Sippel:** Investigation, Writing – review & editing. **Elisabeth J. Leehr:** Writing – review & editing. **Glenn Lawyer:** Software, Writing – review & editing. **Francesco Albasini:** Investigation, Writing – review & editing. **Christian Frischholz:** Investigation, Writing – review & editing. **Rainald Mössner:** Writing – review & editing. **Vanessa Nieratschker:** Writing – review & editing. **Julian Rubel:** Writing – review & editing. **Andreas Fallgatter:** Writing – review & editing. **Ann-Christine Ehlis:** Writing – review & editing, Project administration, Supervision. **David Rosenbaum:** Conceptualization, Methodology, Software, Formal analysis, Investigation, Resources, Data curation, Writing – review & editing, Project administration, Supervision, Visualization, Funding acquisition.

## Declaration of Competing Interest

The authors declare that they have no known competing financial interests or personal relationships that could have appeared to influence the work reported in this paper.

## Data Availability

Data will be made available on request.
