# Supplementary material for: In situ fNIRS measurements during cognitive behavioral emotion regulation training in rumination-focused therapy: A randomized-controlled trial
Source: Neuroimage Clin. 2023 Oct 13;40:103525. doi: 10.1016/j.nicl.2023.103525 (PMC10589893; doi:10.1016/j.nicl.2023.103525)
Supplement: Supplementary data 1 [file mmc1.docx]

Supplementary material:

**In-situ fNIRS measurements during cognitive behavioral emotion regulation training in rumination-focused therapy: a randomized-controlled trial**

Hendrik Laicher ^a, b^, Isabell Int-Veen ^a, b^, Leonie Woloszyn ^a, b^, Ariane Wiegand ^a, b, c^, Agnes Kroczek ^a, b^, Daniel Sippel ^a, b^, Elisabeth J. Leehr ^d^, Glenn Lawyer ^e^, Francesco Albasini ^a, b^, Christian Frischholz ^a, b^, Rainald Mössner ^a, b^, Vanessa Nieratschker ^a, b^, Julian Rubel ^f^, Andreas Fallgatter ^a, b, g^, Ann-Christine Ehlis ^a, b, g^, David Rosenbaum ^a, b^

^a^ Department of Psychiatry and Psychotherapy, University Hospital of Tuebingen, Center for Mental Health, Tuebingen, Germany

^b^ German Center for Mental Health (Deutsches Zentrum für Psychische Gesundheit, DZPG), Tuebingen, Germany

^c^ Max-Planck Institute of Psychiatry, Munich, Germany

^d^ Institute for Translational, University of Muenster, Muenster, Germany

^e^ Machine Learning Solutions, Luxembourg, Luxembourg

^f^ Psychotherapy Research Unit, Department of Psychology, Osnabrueck University, Osnabrueck, Germany

^g^ LEAD Graduate School & Research Network, University of Tuebingen, Tuebingen, Germany

**Corresponding Author:**

Hendrik Laicher, Calwerstraße 14, 72076 Tuebingen, Germany

email: hendrik.laicher@med.uni-tuebingen.de

Phone: 00497071 29-87103

**Keywords:** functional near-infrared spectroscopy (fNIRS), Emotion Regulation, Major Depression Disorder, Psychotherapy, in situ measurements, Repetitive Negative Thinking (RNT)

**Supplementary material**

**Procedure of the TSST**

We adapted the procedure of the original TSST due to the fNIRS-setup by adding resting state measurements and control tasks as we already did in previous studies (Laicher et al., 2022; Rosenbaum et al., 2021; Rosenbaum et al., 2018c). After arriving at the laboratory, participants completed several questionnaires assessing demographic variables and ruminative habits (Rumination Response Scale, RRS Nolen-Hoeksema, 1991) – both only at the first TSST measurement – and further at each measurement: depressive symptom severity (Becks depression inventory II, BDI-II, Hautzinger et al., 2009), self-efficacy (Skala zur Allgemeinen Selbstwirksamkeitserwartung, SWE, Jerusalem & Schwarzer, 2003), self-compassion (The Self-Compassion Scale German Version; SCS-D, Hupfeld & Ruffieux, 2011). Further, momentary rumination was assessed three times during each TSST using a state rumination questionnaire (SRQ) which has also been implemented in our previous studies (Rosenbaum et al., 2017; Rosenbaum et al., 2018a; Rosenbaum et al., 2020; Rosenbaum et al., 2018b; Rosenbaum et al., 2018c); for items and scale statistics see tables S1, S2, S3 and S4. Each TSST measurement took approximately 2 to 2.5 hours. At the end, the psychotherapist of the study checked upon the patient, giving acute stabilization if needed. Like that, every patient furthermore already got to know the psychotherapist after the first TSST (independent of the study group). Patients were debriefed in detail after their last TSST measurement. As the focus of this paper is the analysis of the in-situ measures as well as the psychometric effects of the therapeutic training sessions, we will not go further into detail about the TSST measurements. The data of those will be analyzed in additional papers. However, more detailed information regarding our TSST procedure is to be found in our previous publications (Laicher et al., 2022; Rosenbaum et al., 2021; Rosenbaum et al., 2018c).

Table S1

*State rumination questionnaire (SRQ).*

Im Folgenden werden Ihnen Fragen zu den letzten 10 Minuten gestellt. Wir bitten Sie anzugeben, inwiefern die folgenden Aussagen über ihr Erleben während dieser Zeit übereinstimmen. Sie können hierzu die folgenden Einschätzungen abgeben: „gar nicht“, „fast nicht“, „ein wenig“, „oft“ und „sehr oft“.

|  |  | gar nicht | fast nicht | ein wenig | oft | sehr oft |
| --- | --- | --- | --- | --- | --- | --- |
| 1 | Ich dachte immer wieder an meine Probleme. |  |  |  |  |  |
| 2 | Ich verharrte im Denken an Dinge die mich beunruhigen. |  |  |  |  |  |
| 3 | Meine Gedanken wiederholten sich, ohne dass ich zu einer Lösung kam. |  |  |  |  |  |
| 4 | Ich verlor mich in meinen negativen Gedanken. |  |  |  |  |  |
| 5 | Ich konnte meine Gedanken nur mühsam festhalten. |  |  |  |  |  |
| 6 | Ich konnte mich nicht von meinen negativen Gedanken lösen. |  |  |  |  |  |
| 7 | Ich war bei der Sache. |  |  |  |  |  |
| 8 | Ich dachte darüber nach, warum ich mich in bestimmten Situationen falsch verhalten habe. |  |  |  |  |  |
| 9 | Ich fragte mich, warum ich Probleme habe, die andere nicht haben. |  |  |  |  |  |
| 10 | Ich fragte mich, womit ich meine momentane Lebenssituation verdient habe. |  |  |  |  |  |
| 11 | Ich dachte darüber nach, warum ich die Dinge nicht besser in den Griff bekomme. |  |  |  |  |  |
| 12 | Ich dachte an all meine Defizite und Misserfolge, Macken und Fehler. |  |  |  |  |  |
| 13 | Ich konnte flexibel zwischen meinen Gedanken hin und her schalten |  |  |  |  |  |
| 14 | Ich dachte an vergangene Situationen, die ich bereue. |  |  |  |  |  |
| 15 | Ich machte mir Selbstvorwürfe. |  |  |  |  |  |
| 16 | Ich verlor mich in Gedanken an Vergangenes. |  |  |  |  |  |
| 17 | Ich war von meinen Problemen und Sorgen stark vereinnahmt. |  |  |  |  |  |
| 18 | Meine negativen Gedanken ließen mich nicht los. |  |  |  |  |  |

Table S2

*Cronbach’s Alpha statistics of the state rumination questionnaire (SRQ).*

|  | t_1_ | t_2_ | t_3_ |
| --- | --- | --- | --- |
| SRQ 1^a^ | .953 | .936 | .895 |
| SRQ 2^a^ | .957 | .954 | .880 |
| SRQ 3^a^ | .964 | .948 | .898 |

*Note.* ^a^SRQ 1 was assessed after an initial resting state measurement without any specific task for the subjects; SRQ 2 was assessed immediately after the stress induction via the TSST; SRQ 3 was assessed after a recovery phase 45 minutes after the stress induction.

Table S3

*Retest reliabilities of the state rumination questionnaire (SRQ).*

|  | Within each measurement | | | |  | Between the measurements | | | |
| --- | --- | --- | --- | --- | --- | --- | --- | --- | --- |
|  | All subjects (t_1_)^b^ | t_1_ | t_2_ | t_3_ |  |  | t_1_ -> t_2_ | t_1_ -> t_3_ | t_2_ -> t_3_ |
| SRQ 1^a^ -> SRQ 2^a^ | .754** | .434** | .773** | .685** |  | SRQ 1^a^ | .300* | .192 | .357* |
| SRQ 1^a^ -> SRQ 3^a^ | .795** | .524** | .760** | .544** |  | SRQ 2^a^ | .351* | .153 | .240 |
| SRQ 2^a^ -> SRQ 3^a^ | .886** | .756** | .883** | .790** |  | SRQ 3^a^ | .370** | .024 | .336* |

*Note.* ^a^SRQ 1 was assessed after an initial resting state measurement without any specific task for the subjects; SRQ 2 was assessed immediately after the stress induction via the TSST; SRQ 3 was assessed after a recovery phase 45 minutes after the stress induction. ^b^In this column the data of depressed patients as well as healthy controls was considered whereas in all other columns only the data of the depressed sample was considered as only these participated in more than one TSST measurement. **p* < .05, ***p* < .01.

Table S4

*Correlations of the state rumination questionnaire (SRQ) with the BDI-II (Hautzinger et al., 2009) and the RRS (Rumination Response Scale; Nolen-Hoeksema, 1991).*

|  | t_1_ | | | t_2_ | | | t_3_ | | |
| --- | --- | --- | --- | --- | --- | --- | --- | --- | --- |
|  | SRQ 1^a^ | SRQ 2^a^ | SRQ 3^a^ | SRQ 1^a^ | SRQ 2^a^ | SRQ 3^a^ | SRQ 1^a^ | SRQ 2^a^ | SRQ 3^a^ |
| BDI-II^b^ | .382** | .433** | .473** | .664** | .681** | .752** | .385** | .461** | .382** |
| BDI-II (all subjects)^d^ | .814** | .783** | .816** |  |  |  |  |  |  |
| RRS^c^ | .389** | .171 | .435** | .260* | .251 | .307* | .324* | .265* | .312* |
| RRS (all subjects)^d^ | .714** | .749** | .779** |  |  |  |  |  |  |

*Note.* ^a^SRQ 1 was assessed after an initial resting state measurement without any specific task for the subjects; SRQ 2 was assessed immediately after the stress induction via the TSST; SRQ 3 was assessed after a recovery phase 45 minutes after the stress induction. ^b^For the correlations with the BDI-II, the BDI-II-scores of the corresponding measurement (t_1_, t_2_, t_3_) was used. ^c^The RRS was only assessed once, namely at t_1_. ^d^In these rows the data of depressed patients as well as healthy controls was considered whereas in the other rows only the data of the depressed sample was considered as only these participated in more than one TSST measurement **p* < .05, ***p* < .01.

Figure S1. fNIRS probeset placement and associated Brodmann areas.


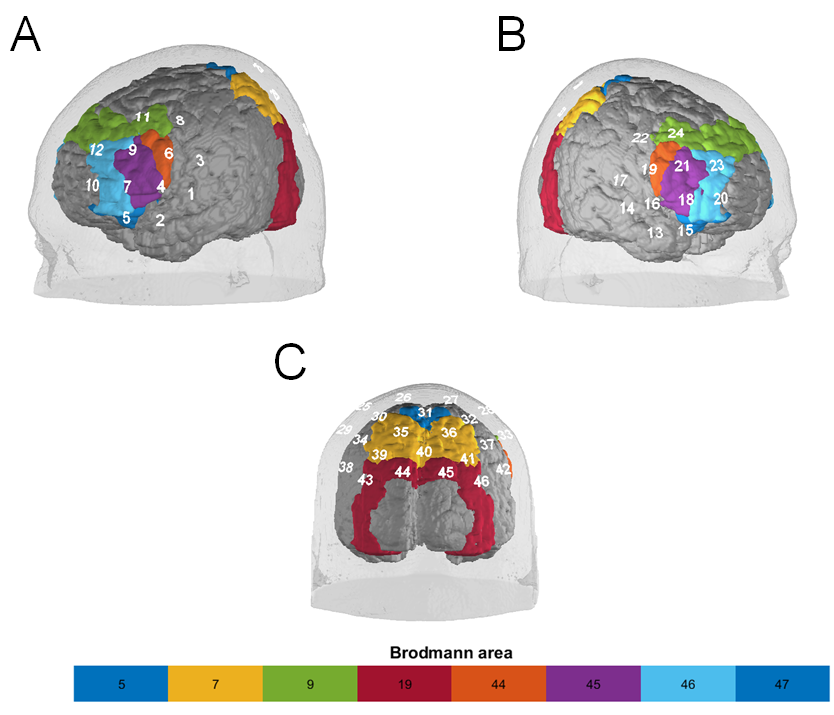


*Note.* A = left frontal probeset, B = right frontal probeset, C = parietal probeset. For probeset placement see also table 2.

Table S5

*Descriptive statistics of the analyzed questionnaires: Mean scores and standard deviations dependent on group and time point of measurement.*

|  | t_1_ | | | | t_2_ | | | | t_3_ | | | |
| --- | --- | --- | --- | --- | --- | --- | --- | --- | --- | --- | --- | --- |
|  | Treatment (*n* = 20) | | TAU (*n* = 21) | | Treatment (*n* = 20) | | TAU (*n* = 21) | | Treatment (*n* = 20) | | TAU (*n* = 21) | |
|  | *M* | *SD* | *M* | *SD* | *M* | *SD* | *M* | *SD* | *M* | *SD* | *M* | *SD* |
| BDI-II | 27.58 | 8.85 | 25.57 | 6.90 | 14.03 | 9.94 | 21.10 | 9.49 | 13.68 | 9.40 | 10.19 | 8.70 |
| SWE | 23.35 | 3.90 | 24.79 | 5.47 | 26.85 | 4.51 | 23.43 | 4.81 | 26.50 | 4.79 | 29.52 | 4.13 |
| SCS | 2.45 | 0.40 | 2.12 | 0.27 | 3.10 | 0.48 | 2.22 | 0.49 | 3.14 | 0.51 | 3.43 | 0.55 |
| SRQ | 3.00 | 0.74 | 3.10 | 0.61 | 1.99 | 0.63 | 2.58 | 0.80 | 1.96 | 0.46 | 1.67 | 0.39 |

Table S6

Results of the mixed models exploring the association between the subjective ratings of subjective burden, self-compassion, equanimity and effort, cortical oxygenation in the different ROIs, sessions and session phases of the training trials (Session:Phase:O_2_Hb). AIC = Akaike Information Criterion; BIC = Bayesian-Information-Criterion; R^2^ = variance explained by the fixed effects. Significant results are shaded whereas darker shadows indicate smaller *p*-values. #*p* < .1, **p* < .05, ***p* < .01, ****p* < .001.

| Dependent Variable | Subjective burden | | | Self-compassion | | | Equanimity | | | Effort | | |
| --- | --- | --- | --- | --- | --- | --- | --- | --- | --- | --- | --- | --- |
|  | Bilateral DLPFC | Bilateral IFG | SAC | Bilateral DLPFC | Bilateral IFG | SAC | Bilateral DLPFC | Bilateral IFG | SAC | Bilateral DLPFC | Bilateral IFG | SAC |
| Intercept | 6.064*** (0.176) | 5.982*** (0.173) | 6.104*** (0.176) | 4.283*** (0.151) | 4.342*** (0.147) | 4.267*** (0.150) | 3.927*** (0.151) | 3.966*** (0.147) | 3.885*** (0.150) | 5.836*** (0.200) | 5.698*** (0.198) | 5.853*** (0.200) |
| Session | -0.143*** (0.024) | -0.126*** (0.023) | -0.149*** (0.025) | 0.244*** (0.022) | 0.240*** (0.021) | 0.242*** (0.023) | 0.199*** (0.023) | 0.195*** (0.022) | 0.204*** (0.024) | -0.267*** (0.023) | -0.239*** (0.022) | -0.270*** (0.024) |
| Phase | -1.311*** (0.078) | -1.254*** (0.074) | -1.312*** (0.077) | 1.328*** (0.072) | 1.309*** (0.068) | 1.331*** (0.072) | 1.316*** (0.075) | 1.288*** (0.071) | 1.316*** (0.075) | -0.872*** (0.075) | -0.782*** (0.071) | -0.850*** (0.074) |
| O_2_Hb | -0.474* (0.230) | -0.317 (0.256) | -0.707** (0.235) | 0.308 (0.213) | 0.255 (0.237) | 0.422# (0.218) | 0.216 (0.221) | 0.132 (0.246) | 0.485* (0.226) | -0.754*** (0.221) | -0.705** (0.247) | -0.840*** (0.226) |
| Session:Phase | 0.025 (0.018) | 0.018 (0.018) | 0.022 (0.019) | -0.057*** (0.017) | -0.057*** (0.016) | -0.053** (0.017) | -0.045* (0.018) | -0.042* (0.017) | -0.044* (0.018) | 0.063*** (0.018) | 0.045** (0.017) | 0.055** (0.018) |
| Session:O_2_Hb | 0.120* (0.058) | 10118 (0.061) | 0.145** (0.056) | -0.020 (0.054) | -0.001 (0.057) | -0.031 (0.051) | -0.038 (0.056) | 0.037 (0.059) | -0.076 (0.054) | 0.157** (0.056) | 0.153* (0.059) | 0.165** (0.053) |
| Phase:O_2_Hb | 0.289# (0.172) | 0.378* (0.181) | 0.314# (0.161) | -0.083 (0.159) | -0.016 (0.168) | -0.122 (0.149) | -0.118 (0.166) | -0.277 (0.174) | -0.152 (0.155) | 0.456** (0.165) | 0.546** (0.175) | 0.340* (0.155) |
| Session:Phase:   O_2_Hb | -0.042 (0.045) | -0.028 (0.045) | -0.033 (0.041) | 0.0003 (0.042) | 0.013 (0.041) | -0.010 (0.038) | 0.012 (0.043) | 0.017 (0.043) | 0.020 (0.039) | -0.083# (0.043) | -0.125** (0.043) | -0.042 (0.039) |
| AIC | 3087.8 | 3087.1 | 3082.2 | 2921.3 | 2922.4 | 2917.7 | 2998.8 | 2992.3 | 2993.7 | 3027.4 | 3029.3 | 3021.7 |
| BIC | 3136.9 | 3136.2 | 3131.4 | 2970.5 | 2971.6 | 2966.9 | 3048.0 | 3041.4 | 3042.8 | 3076.6 | 3078.4 | 3070.9 |
| R^2^ | .346 | .345 | .348 | .404 | .403 | .407 | .392 | .395 | .396 | .174 | .173 | .177 |

Table S7

Results of the mixed models exploring the association between the subjective ratings of subjective burden, self-compassion, equanimity and effort, cortical oxygenation in the different ROIs, sessions and session phases of the training trials (Session:Phase:WP_O_2_Hb + Session:Phase:BP_O_2_Hb). AIC = Akaike Information Criterion; BIC = Bayesian-Information-Criterion; R^2^ = variance explained by the fixed effects. Significant results are shaded whereas darker shadows indicate smaller *p*-values. #*p* < .1, **p* < .05, ***p* < .01, ****p* < .001.

| Dependent Variable | Subjective burden | | | Self-compassion | | | Equanimity | | | Effort | | |
| --- | --- | --- | --- | --- | --- | --- | --- | --- | --- | --- | --- | --- |
|  | Bilateral DLPFC | Bilateral IFG | SAC | Bilateral DLPFC | Bilateral IFG | SAC | Bilateral DLPFC | Bilateral IFG | SAC | Bilateral DLPFC | Bilateral IFG | SAC |
| Intercept | 5.899*** (0.203) | 5.968*** (0.174) | 5.856*** (0.224) | 4.311*** (0.181) | 4.352*** (0.148) | 4.268*** (0.198) | 3.893*** (0.181) | 3.986*** (0.148) | 3.883*** (0.196) | 5.590*** (0.236) | 5.734*** (0.200) | 5.759*** (0.265) |
| Session | -0.163*** (0.028) | -0.124*** (0.023) | -0.169*** (0.031) | 0.258*** (0.036) | 0.236*** (0.021) | 0.231*** (0.028) | 0.197*** (0.027) | 0.194*** (0.022) | 0.187*** (0.030) | -0.266*** (0.027) | -0.244*** (0.022) | -0.279*** (0.030) |
| Phase | -1.316*** (0.090) | -1.240*** (0.074) | -1.290*** (0.100) | 1.304*** (0.084) | 1.289*** (0.069) | 1.290*** (0.093) | 1.279*** (0.087) | 1.266*** (0.071) | 1.220*** (0.096) | -0.879*** (0.087) | -0.782*** (0.071) | -0.896*** (0.096) |
| WP_O_2_Hb | -0.286 (0.274) | -0.094 (0.289) | -0.613* (0.268) | 0.276 (0.253) | 0.015 (0.268) | 0.522* (0.249) | 0.307 (0.264) | -0.015 (0.277) | 0.676** (0.259) | -0.816** (0.264) | -0.390*** (0.278) | -0.756** (0.259) |
| BP_O_2_Hb | 0.630 (0.789) | -0.731 (0.762) | 0.542 (0.750) | 0.101 (0.706) | 0.511 (0.648) | 0.444 (0.663) | 0.434 (0.704) | 0.692 (0.647) | 0.542 (0.657) | 0.884 (0.920) | 0.267 (0.872) | -0.349 (.888) |
| Session:Phase | 0.024 (0.022) | 0.017 (0.018) | 0.019 (0.024) | -0.060** (0.020) | -0.053** (0.016) | -0.047* (0.022) | -0.044* (0.021) | -0.039* (0.017) | -0.034 (0.023) | 0.068** (0.021) | 0.045** (0.017) | 0.062** (.023) |
| Session:WP_O_2_Hb | 0.066 (0.068) | -0.017 (0.072) | 0.105 (0.065) | 0.012 (0.063) | 0.055 (0.067) | -0.051 (0.060) | -0.045 (0.066) | 0.043 (0.069) | -0.104# (0.062) | 0.162* (0.066) | 0.226** (0.070) | 0.151* (0.062) |
| Phase:WP_O_2_Hb | 0.252 (0.202) | 0.218 (0.216) | 0.336# (0.190) | -0.133 (0.187) | 0.076 (0.200) | -0.192 (0.177) | -0.207 (0.195) | -0.029 (0.207) | -0.311# (0.183) | 0.454* (0.195) | 0.536** (0.208) | 0.265 (0.183) |
| Session:BP_O_2_Hb | 0.246* (0.109) | 0.070 (0.100) | 0.242* (0.103) | -0.103 (0.101) | -0.126 (0.093) | 0.020 (0.095) | -0.024 (0.105) | 0.001 (0.096) | -0.003 (0.099) | 0.151 (0.105) | 0.013 (0.096) | 0.204* (0.099) |
| Phase:BP_O_2_Hb | 0.314 (0.353) | 0.730* (0.342) | 0.208 (0.334) | 0.096 (0.327) | -0.696* (0.301) | 0.066 (0.310) | 0.136 (0.341) | -0.838** (0.312) | 0.290 (0.322) | 0.500 (0.340) | 0.560# (0.312) | 0.551# (0.322) |
| Session:Phase:  WP_O_2_Hb | -0.036 (0.051) | -0.021 (0.052) | -0.034 (0.047) | -0.005 (0.047) | -0.034 (0.048) | 0.0001 (0.044) | 0.018 (0.049) | -0.019 (0.050) | 0.036 (0.046) | -0.075 (0.049) | -0.129* (0.050) | -0.032 (0.045) |
| Session:Phase:  BP_O_2_Hb | -0.033 (0.085) | -0.042 (0.077) | -0.020 (0.080) | 0.009 (0.079) | 0.121# (0.072) | -0.036 (0.074) | 0.001 (0.082) | 0.107 (0.074) | -0.026 (0.077) | -0.114 (0.082) | -0.123# (0.074) | -0.071 (0.077) |
| AIC | 3084.6 | 3089.8 | 3080.5 | 2925.4 | 2925.2 | 2924.6 | 3004.3 | 2994.0 | 2996.2 | 3031.2 | 3028.8 | 3028.2 |
| BIC | 3153.4 | 3158.6 | 3149.4 | 2994.2 | 2994.0 | 2993.4 | 3073.1 | 3062.8 | 3065.0 | 3100.0 | 3097.6 | 3097.0 |
| R^2^ | .388 | .348 | .386 | .406 | .406 | .411 | .396 | .398 | .406 | .208 | .179 | .185 |

What triggers rumination in me?

In the last session, you already got to know the model of how ruminative processes occur. For each person, the emotions that trigger rumination can be completely different. Therefore, it is important that you become aware of your own critical emotional states as a result of which you are ruminating.

**
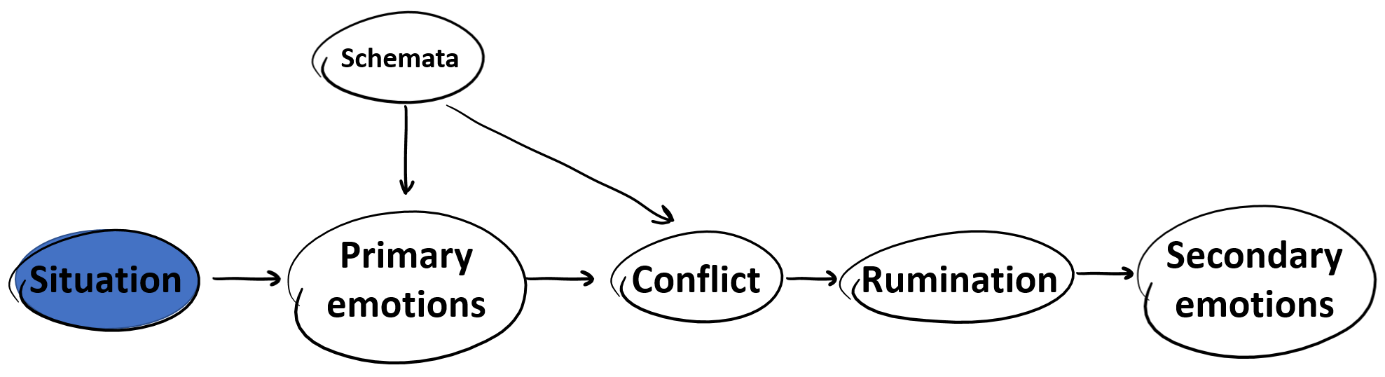
**In the following, you should get to know yourself better by answering some questions for yourself in writing.

1. **Identifying situations that trigger rumination**

First, think of a situation in which you last ruminated. A situation in which your thoughts were stuck in the past and you had to think about something over and over again, although you did not come to a solution. Do similar situations occur more often where you also ruminated? What situations typically trigger ruminative processes for you?

|  |
| --- |
|  |
|  |
|  |
|  |
|  |
|  |
| 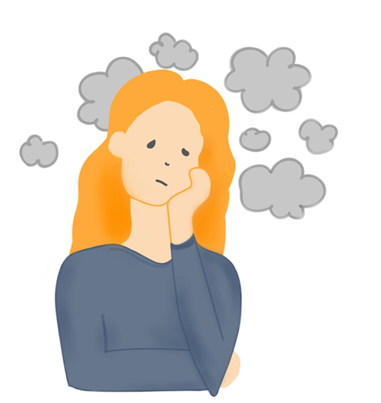 |
|  |
|  |

1.
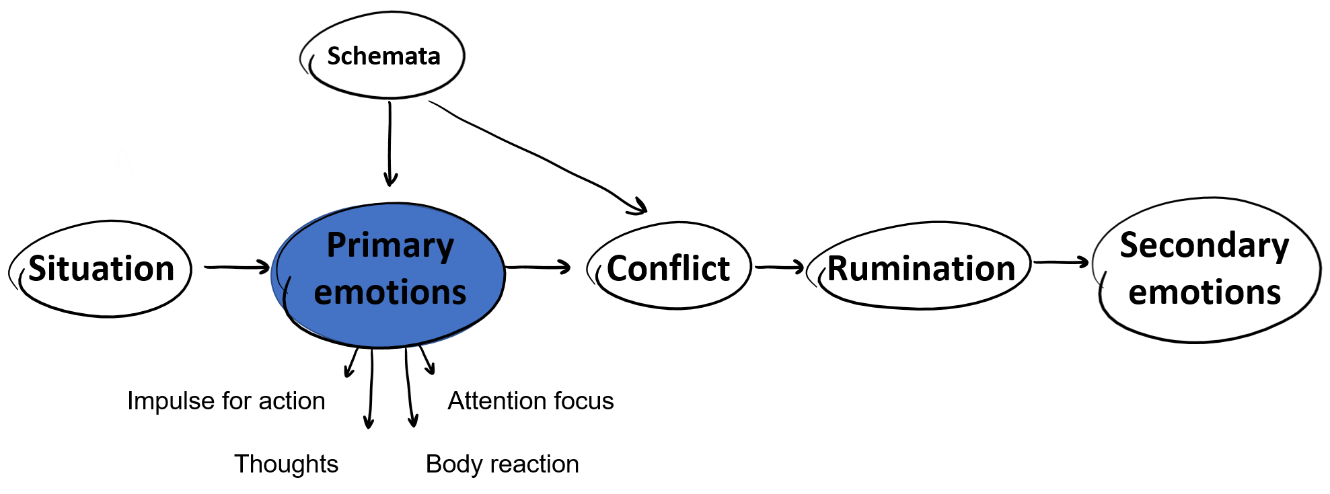
**Identifying emotions that occur as a result of these situations**

When you think of the situations as a result of which you typically ruminate, what emotions were triggered in them? What did you feel and how did it make you feel? Write down which emotion(s) you can name or try to paraphrase.

|  |
| --- |
|  |
|  |

Each emotion expresses itself in different ways: An impulse to act, certain thoughts, a typical body reaction, and often a shift of attention to stimuli that "support" the emotion. Together, this is called the "emotional network." Which do you notice when the triggering emotion occurs? Make one emotional network for each triggering emotion you listed above. Feel free to use the mind map above as a guide and name a few keywords for each point.


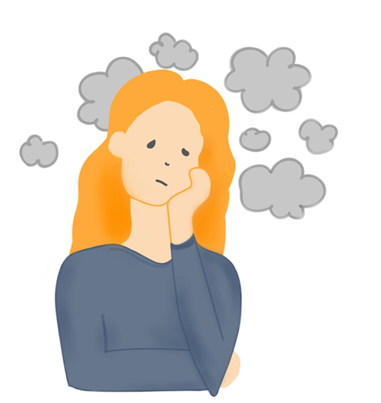


1. **Knowing formative life events and basic beliefs**


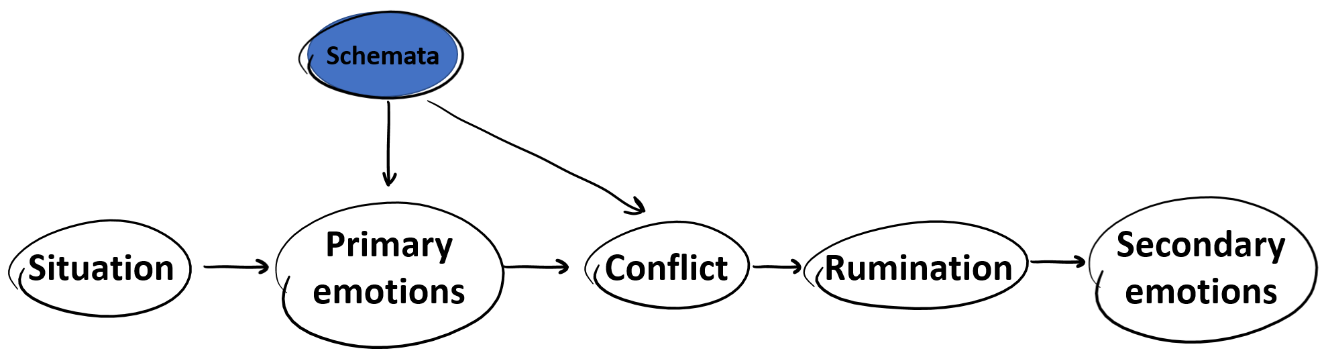


People react emotionally differently in certain situations. What emotions we have depends on our accumulated life experiences. In the course of life, we make different experiences and collect different knowledge about the world: this knowledge is called schema. Certain schemata, meaning basic assumptions, motives, needs or values, determine how we deal with emotions. In order to understand one's own emotions and how to deal with them, it is important to know one's own schemata (e.g. such a schema/belief regarding failure could be "Performance: I must always give my best and must not fail”). It can be helpful to ask oneself: Which formative life events and beliefs (e.g. I must not show weakness) do I have?

|  |
| --- |
|  |
|  |
|  |
|  |
|  |
|  |
|  |
|  |
| 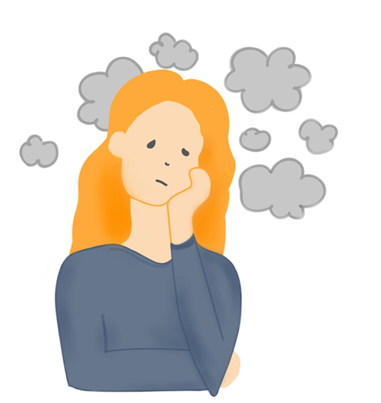 |
|  |
|  |

1. **Summary**


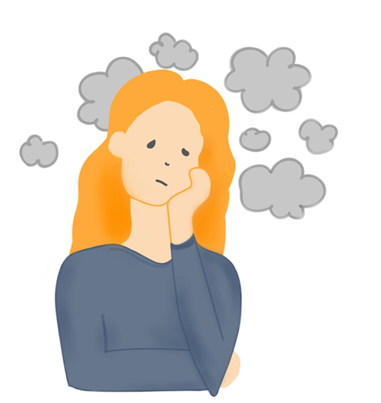
On the following page you will find an "empty" rumination process. In the empty mind map, enter the situations that typically trigger rumination for you, the emotions associated with them, and your own personal schemata (needs, motives, values, and basic assumptions) to record what you learned today.


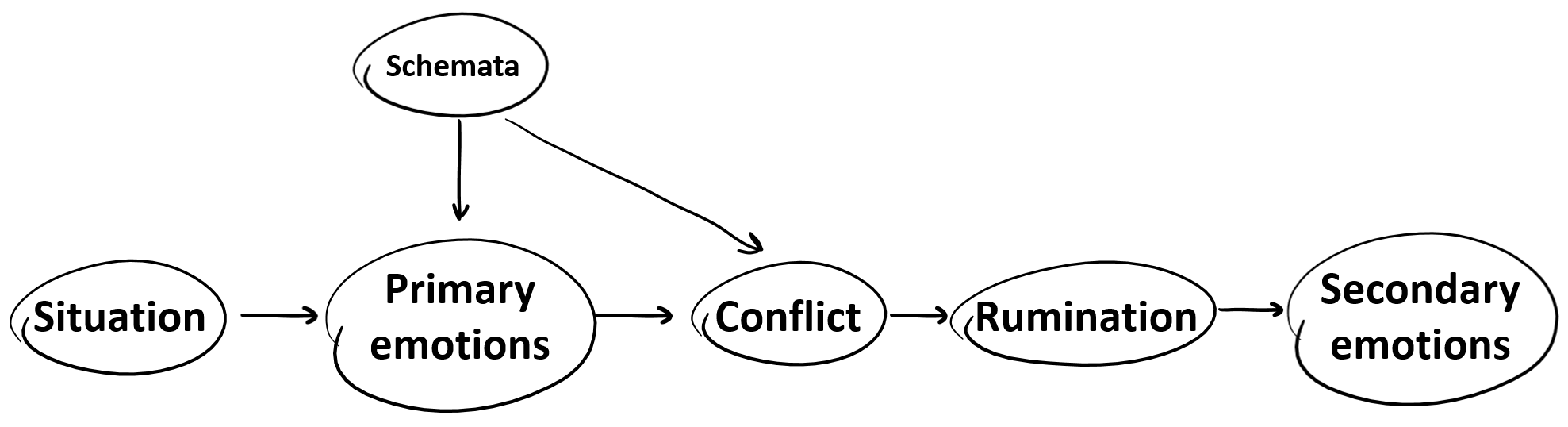


How can I deal with emotions?

We have already learned that conflicts in dealing with emotions can lead to rumination. In doing so, we have identified situations and associated feelings that trigger a typical reaction due to certain beliefs. In order to avoid such conflicts and to learn to deal with our emotions, we can become aware of how to deal with them in a meaningful way:

The prerequisite for this is always that we are not in an acute crisis. For example, on a scale of 1 to 100, we would not indicate a stress level above 50.

| 1 | **Focus**  |
| --- | --- |
|  | We focus on the here and now,  by closing our eyes and consciously  perceive the critical feeling. |
|  |  |
| 2 | **Acceptance** |
|  | We allow ourselves to feel this way by focusing on  on what exactly the feeling is doing to us.  In doing so, we tell ourselves: It is normal that ... |
|  |  |
| 3 | **Self-compassion** |
|  | We meet ourselves with self-compassion.  In doing so, we say to ourselves: Even if I  feel like this, I know that ... |
|  |  |
| 4 | **Distancing** |
|  | We react calmly to these impressions  and let go. |
|  |  |
| 5 | **Calming** |
|  | We can slowly calm down. |


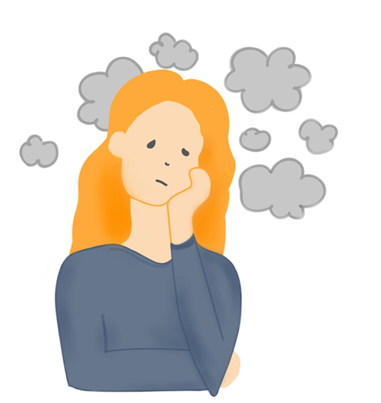
This cycle repeats itself over and over again. With calming down, we focus again on our sensations and on what the feeling does with us.

To make this process more concrete, it is helpful to think about exactly how the individual steps can look. For this, think of exercises or sentences that are as simple as possible so that you can remember them well and perform them at any time. In the following illustration, you have the possibility to note some ideas.

| 1 | **Focus** |
| --- | --- |
|  | We focus on the here and now by ... |
|  |  |
| 2 | **Acceptance** |
|  | We allow ourselves to feel that way by ... |
|  |  |
| 3 | **Self-compassion** |
|  | We meet ourselves with self-compassion.  In doing so, we say to ourselves: ... |
|  |  |
| 4 | **Distancing** |
|  | We react calmly to these impressions  and let go. |
|  |  |
| 5 | **Calming** |
|  | We can slowly calm down. |

**Rumination: Analysis**

Ruminative thought & process: ____________________________________________________________________________________________________________________________________________________


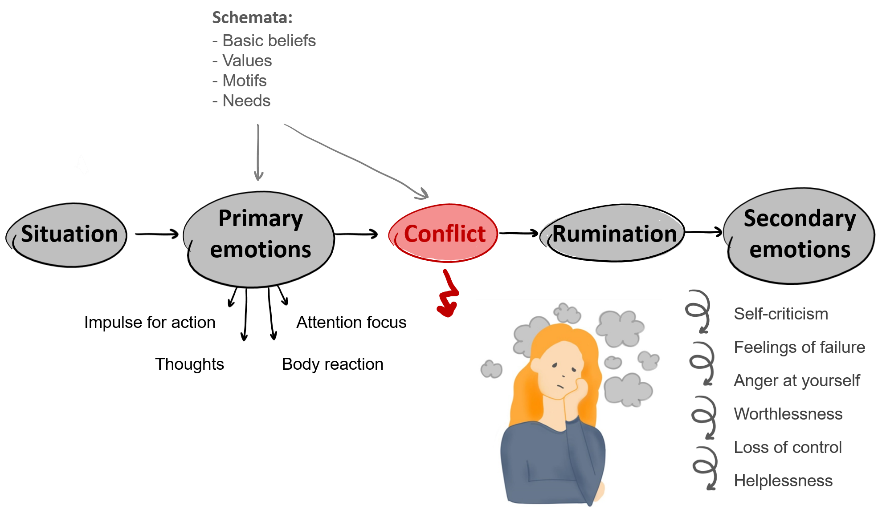


What **basic belief (schema)** might underlie the rumination and my emotions in the situation? (Example: "I must not make mistakes.") ____________________________________________________________________________________________________________________________________________________

What (primary) emotion(s) underlie this rumination?

____________________________________________________________________________________________________________________________________________________

Rumination is often triggered by **inner conflicts**, which are triggered by our motivation "to be safe" (security) and/or "to achieve something" (approach).

Security - 0 to 100 how *un*safe did you feel in the situation: ____________________________

Security - 0 to 100 how much security would you like to have: __________________________

Approach - 0 to 100 how much did you feel motivated to achieve something: ______________

Approach - 0 to 100 how much did you wish / how important was it for you to achieve something: ____________

Please try to describe your inner conflict:

**End the rumination:**

1. **Focus**: I consciously deal with the emotion.

What am I feeling right now? How is my body reacting? Where is the emotion coming from?
Consciously notice your breath, sensations, thoughts and emotions without dwelling on or following them.

Now become aware of how you have dealt with the emotions so far and how you actually want to deal with the emotions and yourself (example: "I am afraid of making a mistake and start ruminating. I don't want to put myself down now and dwell on the situation, but be kind to myself and respond calmly.")

__________________________________________________________________________________________________________________________________________________________________________________________________________________________________________________________________________________________________________________________________________________________________________________________________________________________________________________

1. **Allow and normalize the emotion(s)**: I am allowed to have this (these) emotion(s). Anyone would feel that way. (Example: "Any person would be afraid of doing something wrong in an important task"). __________________________________________________________________________________________________________________________________________________________________________________________________________________________________________________________________________________________________________________________________________________________________________________________________________________________________________________
2. **Reframe the emotion(s):** Change the perspective on the emotion(s). Be benevolent and compassionate with yourself. (Example: "Even though I am afraid of making a mistake now, I know that I am not defined by my mistakes. Regardless of my mistakes, I am a valuable person.") Try to find the reinterpretations that fit for you.

____________________________________________________________________________________________________________________________________________________________________________________________________________________________________________________________________________________________________________________________________________________________________________________________________________________________________________________________________________________________________________________________________________________________________________________________________________________________________________________________________________________________________________________________________________________________________________________________________________________________________________________________________________________________________

1. **Distance yourself from the emotion(s):** Be aware of the emotion(s), but don't get caught up in it (them). Stay in the here and now. Imagine looking at the emotion(s) from a distance. Close with the conflict for the moment and focus on the here and now. Then: Take concrete action according to your goals.

_________________________________________________________________________________________________________________________________________________________________________________________________________________________________________________________________________________________________________________________________________________________________________________________________________________________________________________________________________________________________________________________________________________________________________________________________________________________________________________________________________________________________________________________________________________________________________________________________________________________________________________________________________________________________________________________________

**Literature**

Hautzinger, M., Keller, F., Kühner, C., & Beck, A. T. (2009). *Beck depressions-Inventar: BDI II; manual*. Pearson Assessment.

Hupfeld, J., & Ruffieux, N. (2011). Validierung einer deutschen version der Self-Compassion Scale (SCS-D). *Zeitschrift für Klinische Psychologie und Psychotherapie*, *40*(2), 115-123.

Jerusalem, M., & Schwarzer, R. (2003). SWE - Skala zur Allgemeinen Selbstwirksamkeitserwartung. <https://doi.org/http://dx.doi.org/10.23668/psycharchives.307>

Laicher, H., Int-Veen, I., Torka, F., Kroczek, A., Bihlmaier, I., Storchak, H., Velten-Schurian, K., Dresler, T., Täglich, R., & Fallgatter, A. J. (2022). Trait rumination and social anxiety separately influence stress-induced rumination and hemodynamic responses. *Scientific Reports*, *12*(1), 1-16.

Nolen-Hoeksema, S. (1991). Responses to depression and their effects on the duration of depressive episodes. *Journal of Abnormal Psychology*, *100*(4), 569-582. <https://doi.org/10.1037/0021-843X.100.4.569>

Rosenbaum, D., Haipt, A., Fuhr, K., Haeussinger, F. B., Metzger, F. G., Nuerk, H.-C., Fallgatter, A. J., Batra, A., & Ehlis, A.-C. (2017). Aberrant functional connectivity in depression as an index of state and trait rumination. *Scientific Reports*, *7*(1), 2174. <https://doi.org/10.1038/s41598-017-02277-z>

Rosenbaum, D., Hilsendegen, P., Thomas, M., Haeussinger, F. B., Nuerk, H.-C., Fallgatter, A. J., Nieratschker, V., Ehlis, A.-C., & Metzger, F. G. (2018). Disrupted prefrontal functional connectivity during post-stress adaption in high ruminators. *Scientific Reports*, *8*(1), 15588. <https://doi.org/10.1038/s41598-018-33777-1>

Rosenbaum, D., Int-Veen, I., Laicher, H., Torka, F., Kroczek, A., Rubel, J., Lawyer, G., Bürger, Z., Bihlmaier, I., & Storchak, H. (2021). Insights from a laboratory and naturalistic investigation on stress, rumination and frontal brain functioning in MDD: An fNIRS study. *Neurobiology of stress*, *15*, 100344.

Rosenbaum, D., Kroczek, A. M., Hudak, J., Rubel, J., Maier, M. J., Sorg, T., Weisbender, L., Goldau, L., Mennin, D., & Fresco, D. M. (2020). Neural correlates of mindful emotion regulation in high and low ruminators. *Scientific Reports*, *10*(1), 1-15.

Rosenbaum, D., Maier, M. J., Hudak, J., Metzger, F. G., Wells, A., Fallgatter, A. J., & Ehlis, A.-C. (2018). Neurophysiological correlates of the attention training technique: A component study. *NeuroImage: Clinical*, *19*, 1018-1024. <https://doi.org/10.1016/j.nicl.2018.06.021>

Rosenbaum, D., Thomas, M., Hilsendegen, P., Metzger, F. G., Haeussinger, F. B., Nuerk, H.-C., Fallgatter, A. J., Nieratschker, V., & Ehlis, A.-C. (2018). Stress-related dysfunction of the right inferior frontal cortex in high ruminators: An fNIRS study. *NeuroImage: Clinical*, *18*, 510-517. <https://doi.org/10.1016/j.nicl.2018.02.022>
